# Biostimulation can prime elicitor induced resistance of grapevine leaves to downy mildew

**DOI:** 10.3389/fpls.2022.998273

**Published:** 2022-11-09

**Authors:** Lucile Jacquens, Sophie Trouvelot, Christelle Lemaitre-Guillier, Yuko Krzyzaniak, Gilles Clément, Sylvie Citerne, Grégory Mouille, Estelle Moreau, Marie-Claire Héloir, Marielle Adrian

**Affiliations:** ^1^ Agroécologie, Institut Agro Dijon, CNRS, INRAE, Univ. Bourgogne, Univ. Bourgogne Franche-Comté, Dijon, France; ^2^ Institut Jean-Pierre Bourgin, INRAE, AgroParisTech, CNRS, Université Paris-Saclay, Versailles, France; ^3^ Laboratoires Goëmar, Parc Technopolitain Atalante, Saint Malo, France

**Keywords:** grapevine, biostimulant, phenotyping, defense elicitor, plant immunity, vitroplants

## Abstract

Using plant defense elicitors to protect crops against diseases is an attractive strategy to reduce chemical pesticide use. However, development of elicitors remains limited because of variable effectiveness in the field. In contrast to fungicides that directly target pathogens, elicitors activate plant immunity, which depends on plant physiological status. Other products, the biostimulants, can improve certain functions of plants. In this study, the objective was to determine whether a biostimulant *via* effects on grapevine physiology could increase effectiveness of a defense elicitor. A new methodology was developed to study biostimulant activity under controlled conditions using *in vitro* plantlets. Both biostimulant and defense elicitor used in the study were plant extracts. When added to the culture medium, the biostimulant accelerated the beginning of plantlet growth and affected the shoot and root development. It also modified metabolomes and phytohormone contents of leaves, stems, and roots. When applied on shoots, the defense elicitor changed metabolite and phytohormone contents, but effects were different depending on whether plantlets were biostimulated or controls. Defense responses and protection against *Plasmopara viticola* (downy mildew agent) were induced only for plantlets previously treated with the biostimulant, Therefore, the biostimulant may act by priming the defense elicitor action. In this study, a new method to screen biostimulants active on grapevine vegetative growth was used to demonstrate that a biostimulant can optimize the efficiency of a plant defense elicitor.

## Introduction

Viticulture is important worldwide, covering approximately 7.3 million hectares (https://www.oiv.int/en/statistiques/recherche), and the global wine market was valued at approximately US$430.99 billion in 2021 (https://www.zionmarketresearch.com/report/wine-market). Despite its importance and value, viticulture must meet several challenges, including reductions in chemical inputs to vineyards and adaptation to the evolving climate. Grapevine is susceptible to cryptogamic diseases such as downy and powdery mildews and must be protected to ensure yields and wine quality. The global pesticides market was estimated to be US$57.00 billion in 2019 (https://www.businesswire.com/news/home/20200814005279/en/Global-Pesticides-Market-Outlook-2019-to-2027—Featuring-BASF-Bayer-DowDuPont-Among-Others—ResearchAndMarkets.com). In many countries worldwide, social demands and policy incentives to reduce the agricultural chemical footprint are increasing, and viticulture is beginning to move toward sustainability with development of ecofriendly practices. For grapevine, integrated pest management has developed tremendously in recent years, together with organic viticulture and biodynamics. Grapevine breeding is also making progress in generating disease-resistant varieties, mainly to downy and powdery mildews (Yobrégat, 2018). The use of plant defense elicitors to induce grapevine resistance to diseases is another strategy under investigation (Walters et al., 2013; Delaunois et al., 2014).

Plant defense elicitors are compounds or microorganisms that trigger plant immunity (Walters et al., 2013). Perception of elicitors by plant cells triggers a cascade of signaling events that include production of active oxygen species, increase in cytosolic calcium concentration, and MAPK phosphorylation. Those events induce activation of defense genes, leading to the synthesis of Pathogenesis-related (PR) proteins and phytoalexins, cell-wall strengthening, and in some cases, hypersensitive reaction (Eckardt, 2017). Induction of such a set of defenses can provide plant resistance against pathogens. Therefore, elicitor-induced resistance has been developed as a crop protection strategy, and several elicitors are now marketed. However, further development of the strategy is difficult because elicitors have variable efficiency. Several factors likely explain such variability, including environmental conditions, cultural practices, and plant genotypes (Walters et al., 2013; Delaunois et al., 2014). Although some have antifungal activity, elicitors are not fungicides. In contrast to fungicides that act directly on pathogens, elicitors have an indirect mode of action and activate plant immunity. Most importantly, elicitors must first penetrate plant tissues in order to be perceived (Paris et al., 2016; Heloir et al., 2019; Abdul Malik et al., 2020). In addition, elicitor efficiency depends on plant immune system performance, which in turn depends on plant physiology. Activating defenses has energy costs that plants fuel through primary metabolism (Bolton, 2009), forcing plants into a trade-off between growth and defense (Neilson et al., 2013; Huot et al., 2014; Havko et al., 2016; Garcia et al., 2021).

Biostimulants are defined as “any substance or microorganism applied to plants with the aim to enhance nutrition efficiency, abiotic stress tolerance and/or crop quality traits, regardless of its nutrients content. By extension, plant biostimulants also designate commercial products containing mixtures of such substances and/or microorganisms” (Du Jardin, 2015). Therefore, biostimulants are not plant elicitors or resistance-inducers against biotic stress or fertilizers. The worldwide biostimulant market is currently developing and is estimated to reach US$4.4 billion in 2025. Because of relevance to agriculture, biostimulants have been the focus of several reviews (e.g., Khan et al., 2009; Du Jardin, 2012; Calvo et al., 2014; Du Jardin, 2015; Yakhin et al., 2017; Drobek et al., 2019; Basile et al., 2020; Rouphael and Colla, 2020a; Rouphael and Colla, 2020b; Del Buono, 2021; Corsi et al., 2022). In viticulture, most field experiments assessed biostimulant activity of seaweed extracts (mainly *Ascophyllum nodosum*), protein hydrolysates, and fulvic or humic acids. Biostimulants improve grapevine development (Khan et al., 2012; Meggio et al., 2020), leaf photosynthetic activity (El-Boray et al., 2015; Salvi et al., 2019; Salvi et al., 2020), fruit set (Gutiérrez-Gamboa et al., 2018), yields and quality of fruit and wine (Khan et al., 2012; Sabir et al., 2014; Sánchez-Gómez et al., 2017; Frioni et al., 2018; Salvi et al., 2019; Taskos et al., 2019; Salvi et al., 2020; Arioli et al., 2021), and also resistance to heat and water stress (Mancuso et al., 2006; Boselli et al., 2019; Bavaresco et al., 2020; Meggio et al., 2020; Irani et al., 2021; Miliordos et al., 2022). For reviews assessing biostimulant studies, see Yilmaz and Gazioglu Şensoy, 2021; Cataldo et al., 2022; Monteiro et al., 2022; and Samuels et al., 2022.

This study investigated whether a biostimulant could increase grapevine response to an elicitor, with the goal to improve the efficiency of elicitor-induced resistance against pathogens. The study used a new method with *in vitro* grapevine plantlets to demonstrate and characterize effects of a biostimulant (a plant extract) on grapevine development and metabolomes. Then, whether biostimulation increased grapevine responsiveness to elicitor treatment and resistance against downy mildew was determined.

## Material and methods

### *In vitro* plantlet culture

*In vitro* plantlets of *Vitis vinifera* L. ‘Marselan’ (a cultivar we routinely use in our lab) were grown in glass culture tubes (24 mm × 200 mm) covered by metal caps containing 15 mL of Murashige and Skoog medium (Murashige and Skoog, 1962) modified as follows: 4.5 mM NH_4_NO_3_, 9.4 mM KNO_3_, 1.1 mM CaCl_2_·2H_2_O, 0.7 mM MgSO_4_·7H_2_O, 0.7 mM KH_2_PO_4_, 18.7 μM H_2_SO_4_, 11.8 μM MnSO_4_·H_2_O, 3 μM KI, 0.3 μM ZnSO_4_·7H_2_O, 0.2 μM NiCl_2_·6H_2_O, 0.2 μM CoCl_2_·6H_2_O, 0.8 μM H_3_BO_3_, 0.2 μM CuSO_4_·5H_2_O, 89 μM NH_4_Fe(SO_4_)_2_·12H_2_O, 0.4 μM biotine, 8 μM nicotinic acid, 5 μM pyridoxine HCl, 3 μM thiamine HCl, 4 μM calcium panthothenate, 100 mg/L myo-inositol, 20 g/L sucrose, 6 g/L HP697 Agar (Duchefa, Haarlem, Netherlands); no hormone added. The medium was adjusted to pH 6.4 ± 0.2 before autoclaving. Plantlets were cultured in a growth chamber with a controlled temperature of 24 ± 2°C and fluorescent light (photosynthetic photon flux density = 40 µmol m^-^²s^-1^; L 30W/77 Fluora; OSRAM, Molsheim, France) with a 16-h light/8-h dark photoperiod. For experiments, one-bud cuttings were prepared from 10-week-old plantlets then transplanted in the culture medium with addition of the biostimulant or water (the control).

### Biostimulant application and sampling

The biostimulant (BS) used in this study was a plant extract (from the aerial parts of a monocotyledon plant) with additions of iron sulfate heptahydrate and zinc sulfate monohydrate (UPL, Mumbai, India). After autoclaving and immediately before distributing the medium in culture tubes, the BS was added to the culture medium at three concentrations: 0.03%, 0.1%, and 0.3% (v/v). Treatments supplemented with BS were coded “BS+”; whereas the control with ultrapure water was coded “BS-”.

Preliminary experiments with 18 plantlets per treatment were conducted in order to determine the most active concentration and to follow the kinetics of plant development. In the following experiments, 168 plantlets were used per treatment (BS+ and BS-). At 4 weeks post treatment (wpt) 120 plantlets were treated with either the defense elicitor or water as the control (60 per treatment), of which 48 were used for analyses (**Supplementary Figure S1**). Leaves, stems, and roots were excised, rinsed in ultrapure water, dried briefly, frozen in liquid nitrogen, and lyophilized for further metabolite and hormone analyses. Three biological repeats were analyzed per experiment.

### Plantlet development

To study shoot development, the following parameters were monitored: date of bud opening (number of days between transplantation and start of bud development), date of the first expanded leaf (number of days between transplantation and the first expanded leaf), shoot height, number of normal-sized leaves (nL), total number of leaves (L), and nL/L ratio. Except for dates of developmental stages, parameters were monitored weekly. Development of the root system was determined by numbers of adventitious and lateral roots. All parameters were monitored for a set of 80 plantlets/treatment.

### Defense elicitor application and sampling

Four-week-old plantlets were treated with a defense elicitor (“DE”). DE is a plant oligosaccharidic elicitor (apple oligopectins, Laboratoires Goëmar/UPL, Saint-Malo, France) that was previously studied on grapevine herbaceous cuttings grown in greenhouses (Krzyzaniak et al., 2018). The DE concentration was determined based on this previous work. Plantlets were totally immersed in either 0.5 g/L DE solution (“DE+”) or ultrapure water (control, “DE-”) for 30 s as described by Farace et al. (2015) and then were placed in a growth chamber. Sixty plantlets were used per treatment (BS+/DE+, BS+/DE-, BS-/DE+, BS-/DE-).

Two days after elicitor or water treatment (DE+ or DE-), 48 plantlets per treatment were dissected and prepared for metabolite and phytohormone analyses. From the remaining 12 plantlets, normally developed leaves were sampled for gene analysis, autofluorescence observation, and induced-resistance assays. Sampled leaves were immediately frozen (-80°C) for gene analysis or placed on wet 3MM paper (Whatman, Velizy-Villacoublay, France) in plastic dishes for autofluorescence observations and induced-resistance assays, as described below.

### Metabolite analysis

Metabolite analysis was performed by gas chromatography mass spectrometry (GC–MS). Lyophilized samples (10 mg), the BS, and the DE were extracted with 0.8 mL of acetone:water:acetic acid (80:19:1 v:v:v) containing ribitol at 4 µg/mL, followed by shaking for 10 min at 4°C at 1400 rpm. After centrifugation (20,000 ×*g*, 5 min), 100 µL of supernatant were collected and dried for 5 h in a SpeedVac vacuum centrifuge.

Analysis and data processing were performed as previously described (Moret et al., 2018). Samples were derivatized and analyzed using an Agilent 7890A gas chromatograph coupled to an Agilent 5975C mass spectrometer, as previously described (Fiehn, 2006; Fiehn et al., 2008). For processing, data files were converted to NetCDF format and analyzed with AMDIS (http://chemdata.nist.gov/mass-spc/amdis/). A homemade retention indices and mass spectra library built from the NIST, Golm (http://gmd.mpimp-golm.mpg.de/), and Fiehn databases and standard compounds was used to identify metabolites. Peak areas were determined with Targetlynx software (Waters) after conversion of the NetCDF file to Masslynx format. AMDIS and Target Lynx in splitless and split 30 mode data were compiled in a single Excel file for comparison. After blank mean subtraction, peak areas were normalized to ribitol and fresh weight.

### Phytohormone analysis

Plant samples were harvested and immediately frozen. Then, samples were ground in liquid nitrogen and freeze-dried. Phytohormones were extracted from 10 mg (dry weight) of tissue with 0.8 mL of acetone:water:acetic acid (80:19:1 v:v:v). The phytohormones salicylic acid (SA), indole-3-acetic acid (IAA), and abscisic acid (ABA) were quantified on a Waters Acquity ultra performance liquid chromatograph coupled to a Waters Xevo Triple quadrupole mass spectrometer TQS (UPLC–ESI–MS/MS) as described previously (Le Roux et al., 2014). One nanogram of each internal standard was added to samples. Compounds were separated on a reverse-phase column (Uptisphere C18 UP3HDO, 100 mm × 2.1 mm, 3 µm particle size; Interchim, Montluçon, France).

### Autofluorescence observations

To visualize accumulation of phenolic compounds (plant defense response), leaves from 4-week-and-2-day-old plantlets were transferred to 0.05% Tween20 solution between a glass slide and coverslip. Tissues were immediately observed using an epifluorescence microscope (Leica Leitz DMRB), (Leica, Wetzlar, Germany) equipped with a filter A (λexc 340/380 nm, λem 425 nm) for UV excitation. Four representative images were taken per leaf using a camera (Nikon DIGITAL SIGHT DS SMc), (Nikon, Japan) with initial resolution of 2560 × 1920 pixels and color depth set at 24 bits. All images were acquired according to the same parameters (gain, time) of exposure in order to compare intensities of autofluorescence. At least three leaves from three distinct plantlets were observed per treatment.

### Defense gene expression

Leaves were excised from nine plantlets per treatment. Extraction of total RNA, DNAse treatment, and reverse transcription were performed as described in Krzyzaniak et al. (2018) for foliar tissues. Relative expression of genes was determined with the 2^-ΔΔCT^ method (Livak and Schmittgen, 2001). The PCR reactions were performed in triplicate, and expression was normalized against that of two reference genes, *EF1α* and *VATP16*, as internal controls. Targeted genes were *PAL* and *STS* (phenylpropanoid pathway), *SAMT1* (salicylic acid signaling), *LOX13* (jasmonic acid (JA) synthesis, JA pathway), and *PR2.1* (beta-1,3-glucanase). Sequences of primer pairs used are reported in **Supplementary Table S1**.

### *Plasmopara viticola* inoculation and protection assays

The *Plasmopara viticola* isolate was maintained on Marselan plants in a greenhouse as previously described by Trouvelot et al. (2008). Sporangia were collected from the lower side of leaves using a brush and suspended in distilled water. The concentration was adjusted to 3.5 × 10^4^ sporangia per milliliter (Deglène-Benbrahim et al., 2010) using an hemocytometer (Malassez cell counting).

Two days after treatment (“DE-” or “DE+”), at least six leaves per treatment were inoculated. Each leaf was covered with six droplets (3 µL each) of the *P. viticola* sporangia suspension. Each droplet was deposited in an interveinal area, symmetrically on each half (left and right) of a leaf. To avoid anoxia of leaf tissues, droplets were removed one night later (i.e., approximately 16 h) using sterile absorbent paper. After 7 days of incubation in a growth chamber, each inoculated leaf was rinsed with 1 mL of 50% ethanol. The number of sporangia was then counted in triplicate using a hemocytometer (Malassez cell counting), and the value was reported per cm² of leaf after estimation of leaf area by using ImageJ software. Before rinsing, leaves were photographed to observe the distribution of sporulation.

### Evaluation of leaf colonization by *Plasmopara viticola*

*Plasmopara viticola* development in leaf tissues was assessed after aniline blue staining as described in Trouvelot et al. (2008). Briefly, sampled leaves were fixed one night in 100% methanol, clarified one night in chloral hydrate solution (1 g/mL), and stained overnight with 0.05% aniline blue (in 0.1 M phosphate buffer, pH 8.0). Leaves were then mounted on microscope slides in the staining solution with the abaxial surface uppermost. Pathogen ingress was observed by epifluorescence microscopy under UV (Λ_exc_ 340 nm, Λ_em_ 380 nm, stop filter LP 430 nm) because mycelium fluoresces blue. At least three leaves from three distinct plantlets previously used for protection assays were observed per treatment.

### Statistical analyses

Each biological experiment was conducted in triplicate. Phenotypic data were analyzed with parametric tests. Data from the three experimental repetitions were pooled and significant differences between samples (BS+ vs BS-) were assessed with Student’s *t*-tests (*P < 0.05, **P < 0.01, and ***P < 0.001).

For GC–MS analysis, data were processed with Perseus software (v1.6.8.0, https://maxquant.net/perseus/). Statistical analysis was applied to all organ sets or on separated organ subsets (three replicates per treatment). After Z-scoring and multivariate ANOVA (*P* < 0.05), hierarchical clustering (HCA) was performed with Pearson correlation. Significant metabolites were determined using two-sided Student’s *t*-tests and permutation-based false discovery rate (FDR) set at 5%. Data from phytohormones analysis, sporangia density determination, and gene expression (differences in relative expression) were analyzed by Kruskall–Wallis nonparametric tests (*P* < 0.05).

## Results

### The biostimulant BS has dose-dependent effects on grapevine plantlet development

Three BS concentrations (0.03%, 0.1%, 0.3%) were first assessed on plantlet development for 6 weeks. Compared with the control, both 0.03% and 0.1% concentrations induced significant earlier “bud opening” and “first expanded leaf” stages, with BS 0.1% advancing the stages by 5 and 6 days, respectively (**Table 1**). Both concentrations also promoted shoot development up to 4 weeks post transplantation (wpt), with significant increases in shoot height and leaf number, compared with the control. However, at 6 wpt, the shoot height was not significantly different for control and BS-treated plantlets, and the number of expanded leaves was significantly higher than the control only for BS 0.1% (**Table 1**). Abnormally small-sized leaves were also observed on a few BS-treated plantlets. BS significantly stimulated the development of the root system, only at 0.1% at 4 wpt. At 0.3%, compared with the control, the BS induced significant earlier “bud opening” and “first expanded leaf” stages by 3 and 4 days, respectively. However, only 33% of the cuttings started growth, and those formed plantlets with inhibited root and shoot development and symptoms of toxicity (data not shown). Thus, the 0.1% BS concentration was most beneficial to root and shoot development and therefore was selected as the concentration for the following experiments.

**Table 1:** Effect of two concentrations of the biostimulant BS on the development of grapevine *in vitro* plantlets.

	Bud opening	First expanded leaf	Shoot height (mm)			Number of expanded leaves			Number of roots		
BS (%)	Number of days		3 wpt	4 wpt	6 wpt	3 wpt	4 wpt	6 wpt	3 wpt	4 wpt	6 wpt
0.03%	-3.7*	-4.7*	2.5*	3.5*	-2.3	0.9*	1.0*	0.4	0.5	0.2	0.3
0.10%	-5.0*	-5.9*	3.3*	4.9*	-1.7	1.1*	1.2*	1.4*	0.6	0.6*	0.5

Micro-cuttings were transplanted in a solid culture medium supplemented with BS (0.03% and 0.1% v/v) or water (as control). The dates of bud opening and first expanded leaf (number of days post transplantation) were determined. The shoot height, number of expanded leaves, and number of adventitious roots were monitored at 3, 4, and 6 weeks post transplantation (wpt). Data correspond to the difference in the number of days (for bud opening and first expanded leaf stages), or values obtained (for shoot height, number of leaves and roots) between BS and water treated plantlets. Significant differences between values (control, BS 0.03%, BS 0.1%; for each parameter) were identified with Kruskall-Wallis test with *P* < 0.05. * indicates significant differences between water (control) and BS treatment. Green and red colors indicate positive and negative effects, respectively, compared to the control.

### The biostimulant BS accelerates beginning of plantlet development and affects the phenotype

**Figure 1:**
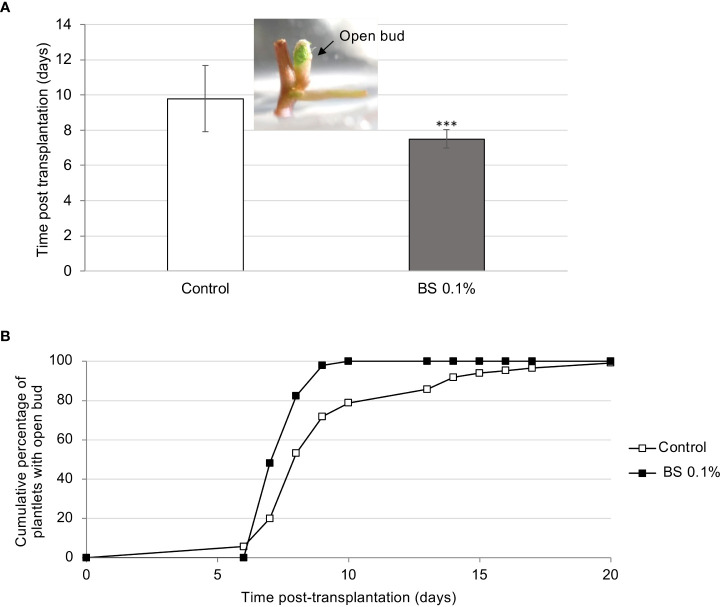
Effect of BS treatment on the beginning of development of *in vitro* grapevine micro-cuttings. Micro-cuttings were transplanted in a solid culture medium supplemented with BS (0.1% v/v) or water (control), and the beginning of their development was compared (“open bud”, with green top of the shoot visible). **(A)** time (mean number of days) between the date of the micro-cutting transplantation and the date of bud opening. **(B)** cumulative percentage of micro-cuttings that have started their development (with open bud). Significant differences were identified with Student *t*-test (***P < 0.001).

The effect of 0.1% BS on plantlet development was examined for 8 wpt. As reported above, the BS significantly accelerated bud opening, compared with the control (7.5 and 9.8 days post transplantation (dpt), for BS+ and control, respectively; **Figure 1A**). Bud opening was also more homogeneous, reaching 100% at 10 dpt for BS-treated plantlets (BS+) compared with 100% only at 20 dpt for control ones (**Figure 1B**). The “first expanded leaf” stage also occurred significantly earlier for BS+ plantlets than for BS- ones (12.4 and 14.7 dpt, respectively; **Supplementary Figure S2**). Moreover, 100% of micro-cuttings developed a plantlet when grown with the BS, whereas 3% of controls did not develop beyond the bud opening stage (**Supplementary Figure S2**).

**Figure 2:**
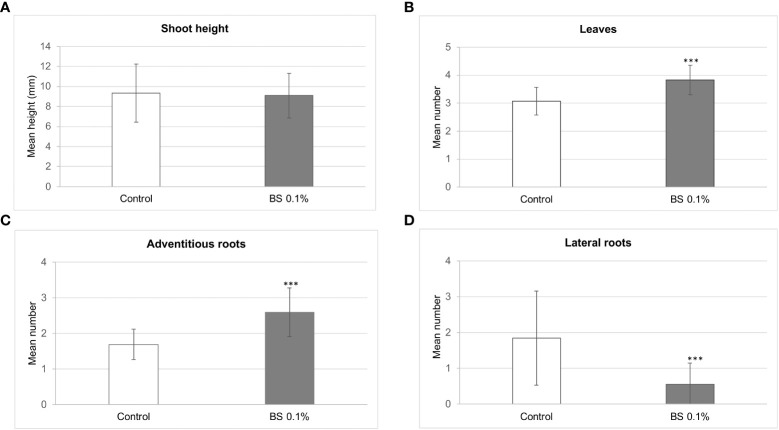
Effect of BS treatment on the development of *in vitro* grapevine plantlets. Micro-cuttings were transplanted in a solid culture medium supplemented with BS (0.1% v/v) or water (as control). The shoot height **(A)**, number of leaves **(B)**, adventitious roots **(C)**, and lateral roots **(D)** was determined at 4 weeks post transplantation (wpt). Significant differences were identified with Student *t*-test (***P <0.001).

The stimulating effects of the BS on shoot development differed according to parameter and time. Height of shoots was greater for BS+ than for BS- plantlets up to 3 wpt, then was not significantly different at 4 wpt, and finally was lower for BS+ than for BS- plantlets (**Table 2**). At 4 wpt, shoot height reached 9.1 and 9.3 mm for BS+ and BS- plantlets, respectively (**Figure 2A**). Because abnormally small leaves were observed on some BS+ shoots, normal-sized (nL) and total leaves (L) were counted, and the nL/L ratio was calculated. Changes in number of normal-sized leaves were similar to changes in shoot height: higher numbers for BS+ than BS- plantlets up to 4 wpt and then not significantly lower. By contrast, total number of leaves remained significantly higher in BS+ than in BS- plantlets from 3 wpt to the end of the experiment (**Table 2**). At 4 wpt, the total number of leaves was 3.8 and 3.1 for BS+ and BS- plantlets, respectively (**Figure 2B**). The nL/L ratio was similar for BS+ and BS- plantlets at 2 and 3 wpt but then was significantly lower for BS+ than for BS- plantlets from 4 wpt (**Table 2**). The BS significantly promoted adventitious root development, whereas it significantly inhibited lateral root development (**Table 2**; **Figures 2C, D**). At 4 wpt, the number of adventitious roots was 2.6 and 1.7 for BS+ and BS- plantlets, respectively (**Figure 2C**). The number of lateral roots was 0.6 and 1.9, respectively (**Figure 2D**).

**Table 2:** Effect of treatment by the biostimulant BS on the development of grapevine *in vitro* plantlets.

	Time post transplantation (weeks)						
	2	3	4	5	6	7	8
Shoot height	1.32***	1.06***	0.08	-1.4	-2.37	-3.4***	-3.72***
Number of leaves (L)	0.71***	0.81***	0.92***	1.28***	1.19***	1.29***	1.33***
Number of normal sized leaves (nL)	0.63***	0.26	0.11	-0.14	-0.21	-0.22	-0.28
nL/L	0.01	-0.09	-0.13***	-0.17***	-0.15***	-0.15***	-0.15***
Number of adventitious roots	0.14	0.35*	0.52***	0.6***	0.68***	0.77***	0.8***

Micro-cuttings were transplanted in a solid culture medium supplemented with BS (0.1% v/v) or water (as control). The shoot height, number of leaves (L), number of normal-sized leaves (nL), and number of adventitious roots were monitored weekly from 2 to 8 weeks post transplantation (wpt). The nL/L ratio was also calculated. Data correspond to the difference between the values obtained for BS and water treated plantlets. The significant differences between these values were identified with Student *t*-test, with ∗*P* < 0.05; ∗∗∗*p* < 0.001. Green and red colors indicate positive and negative effects, respectively, compared to the control.

**Figure 3:**
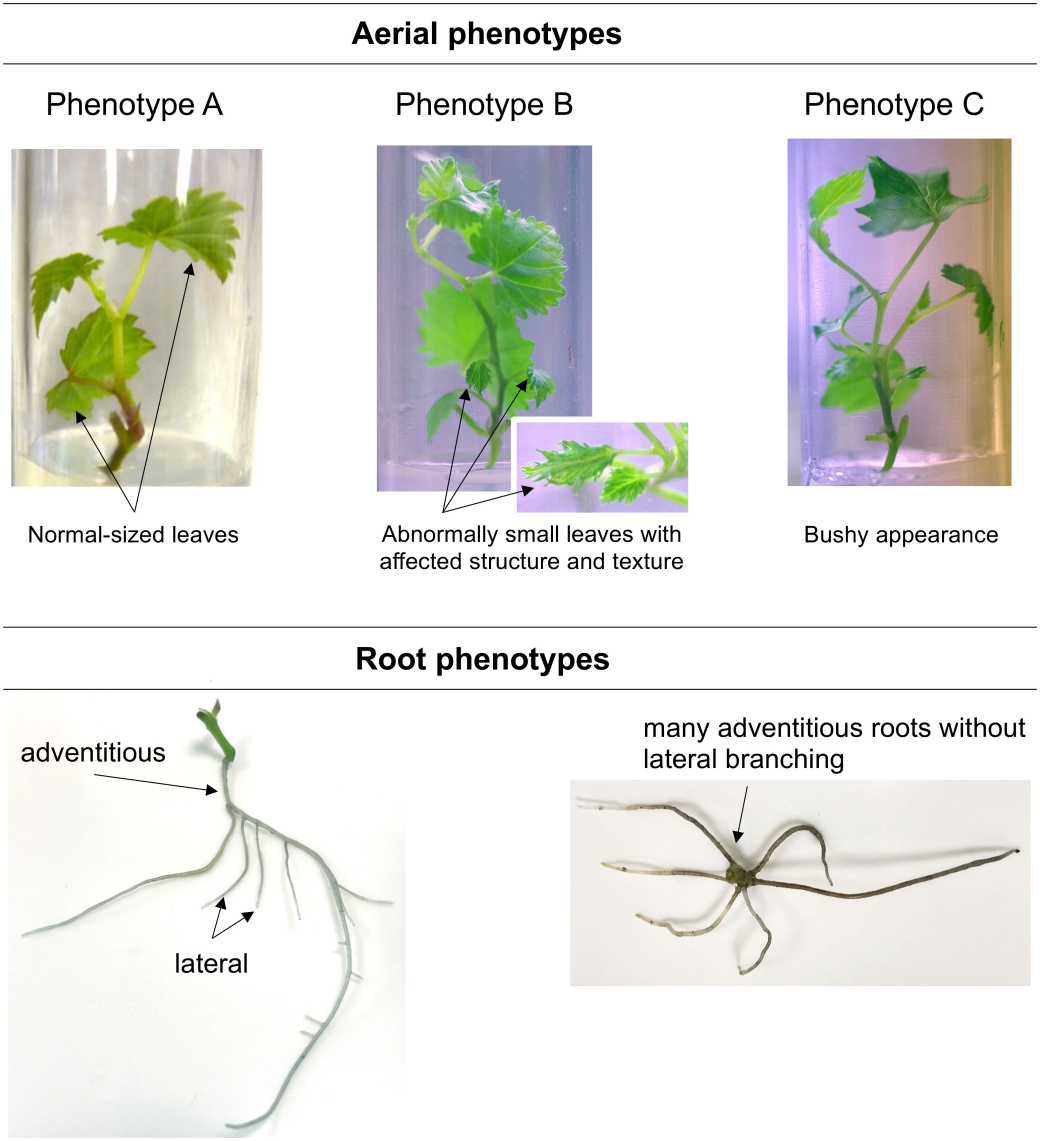
Illustration of the phenotypes observed for control and BS-treated grapevine plantlets. Micro-cuttings were transplanted in a solid culture medium supplemented with BS (0.1% v/v) or water (as control). Their phenotype was observed at 4 wpt. Three aerial phenotypes could be distinguished: shoots with normal-sized leaves (Phenotype A), shoots with the presence of abnormally small leaves (Phenotype B), shoots with bushy appearance (Phenotype C); and two root ones: branched root system with the presence of lateral roots, adventitious roots without lateral branching.

At 4 wpt, three different aerial phenotypes and two distinct root phenotypes were observed (**Figure 3**). Control plantlets developed normal-sized leaves (phenotype A), and the root system was mostly branched, with lateral roots. The BS-treated plantlets showed three aerial phenotypes (**Figure 3**), with unequal distribution: phenotype A (58% of plantlets), a phenotype with some abnormally small leaves with affected structure (phenotype B, 38%), and a phenotype with bushy appearance (branching due to development of some axillary buds; phenotype C, 4%). Moreover, adventitious roots in BS-treated plantlets were mostly unbranched, in contrast to those in the control.

### The biostimulant BS affects the metabolomes and phytohormone contents of plantlet organs

**Figure 4:**
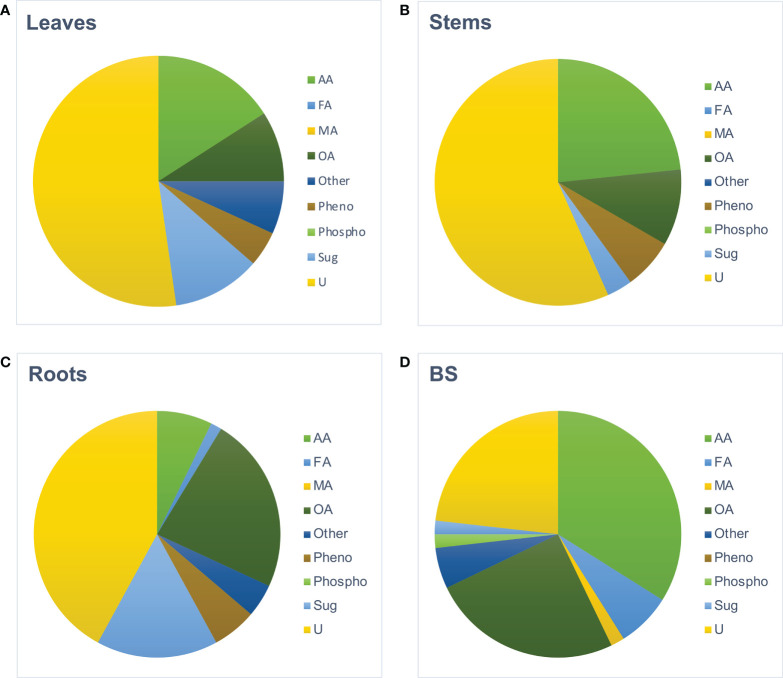
Distribution by biochemical families of the metabolites detected in BS and in the organs of BS-treated grapevine plantlets. Micro-cuttings were transplanted in a solid culture medium supplemented with BS 0.1% (v/v, “BS+”) or water (“BS-”, as control). At 4 wpt, plantlets were collected and dissected to distinct leaf, stem and root samples. Data obtained by GC-MS analysis were processed and the metabolites significantly accumulated in leaves **(A)**, stems **(B)**, and roots **(C)** of BS+ plantlets, compared to BS- ones were categorized as unknown (not annotated) or according to their biochemical family (AA: Amino Acids, FA, Fatty Acids; MA, Mineral Acids; OA, Organic acids; phenol, phenolics *ie* secondary metabolites); Phospho, Phosphorylated compounds; Sug, Sugars; U, unknown). The same categorization was made for compounds detected in the assessed biostimulant **(D)**.

Metabolomes of roots, stems, and leaves from BS+ and BS- plantlets were analyzed at 4 wpt. Hierarchical clustering analysis of all GC–MS data clearly differentiated samples by organ type (data not shown), and thus, data were processed per organ. For each organ, “BS-” and “BS+” samples were distinguished (**Supplementary Figure S3**). Number of metabolites that significantly differentially accumulated was higher for roots (100 metabolites, with 69 increasing and 31 decreasing) than for leaves (49 metabolites, with 43 increasing and 6 decreasing) and stems (35 metabolites, with 31 increasing and 4 decreasing). The distribution of metabolites in biochemical families also varied between organs (**Figure 4**). In BS+ leaves, approximately half of differently accumulated compounds were “unknown” (52%), followed by amino acids (16%), sugars (11%), and organic acids (9%) (**Figure 4A**, percentages not indicated). In stems, the most numerous differently accumulated compounds were also “unknown” (57%), followed by amino acids (23%) and organic acids (10%) (**Figure 4B**, percentages not indicated). In BS+ roots, 42% of differently accumulated compounds were “unknown” (42%), with organic acids (23%) and sugars (16%) the next most represented categories (**Figure 4C**, percentages not indicated). The five most accumulated annotated metabolites in leaves were mannose, pipecolate, piceid, glycosylsalicylate, and gentiobiose (**Supplementary Figure S4**). In stems, valine, leucine, catechin, isoleucine, and threonine were the most accumulated metabolites, and in roots, glucose, mannose, gluconate, salicylate, and threonate were the most accumulated. Composition of the BS was also analyzed for comparison (**Supplementary File 1**). Among 56 detected compounds, 23% were “unknown”, 34% were amino acids, 25% were organic acids, 7% were fatty acids, 5% were “others” (**Figure 4D**, values not indicated). Only nine components were in common with the over-accumulated compounds in BS+ roots (2-oxoglutarate, alanine, erythronate, γ-aminobutyric acid, glycerate, malate, methionine sulfoxide, proline, and threonate (data not shown).

**Figure 5:**
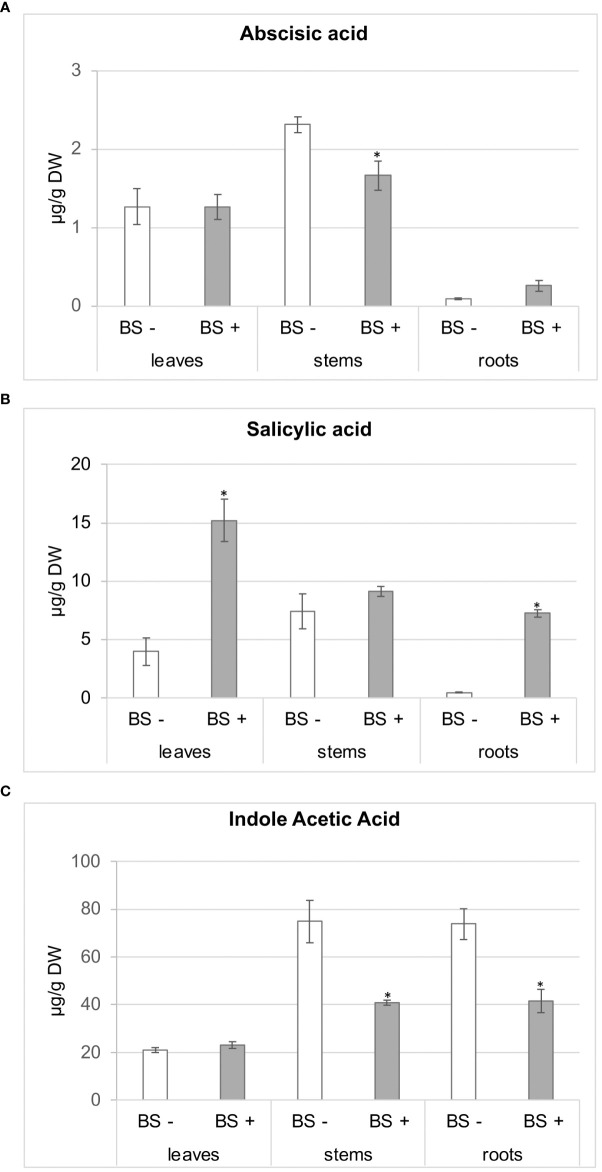
Effect of BS treatment on abscisic acid (ABA), salicylic acid (SA), and indole acetic acid (IAA) contents of grapevine plantlet leaves, stems and roots. Micro-cuttings were transplanted in a solid culture medium supplemented with BS 0.1% (v/v, “BS+”) or water (“BS-”, as control). At 4 wpt, plantlets were collected and dissected to distinct leaf, stem and root samples. Extracts were prepared, and ABA **(A)**, SA **(B)**, IAA **(C)** were analyzed by LC-MS. Significant differences were identified with Kruskall-Wallis test with *P < 0.05.

Regarding phytohormones (**Figure 5**), abscisic acid (ABA) accumulated in similar amounts in leaves of BS- and BS+ plantlets (approximately 1.3 µg/g dry weight (DW)), whereas amounts of ABA in stems were significantly lower in BS+ plantlets than in BS- ones (1.66 and 2.31 µg/g DW, respectively). There were no significant differences in ABA in roots (0.26 and 0.09 µg/g DW for BS+ and BS- plantlets, respectively) (**Figure 5A**). Although there was variability between replicates, salicylic acid (SA) concentrations in leaves and roots were significantly higher in BS+ plantlets than in BS- ones (15.18 and 3.95 µg/g DW for leaves and 7.23 and 0.44 µg/g DW for roots, respectively). Amounts were similar in stems (**Figure 5B**). Concentrations of SA and ABA in BS- plantlets were higher in stems than in leaves, which had higher concentrations than roots. Amounts of the auxin indole acetic acid (IAA) in stems and roots were significantly lower in BS+ plantlets than in BS- ones (74.90 and 40.90 ng/g DW for stems and 73.91 and 41.62 ng/g DW for roots, respectively). Amounts were similar in leaves (21.02 and 23.10 ng/g DW, respectively) (**Figure 5C**). Analysis of the BS indicated that it contained smaller amounts of SA (4 ng/g DW) and ABA (8 ng/g DW) than those detected in plantlets. The addition of the BS to the culture medium provided approximately 0.04 ng of ABA and 21 ng of SA.

### Effects of the defense elicitor on metabolomes and phytohormone contents are different for control and biostimulated plantlets

**Figure 6:**
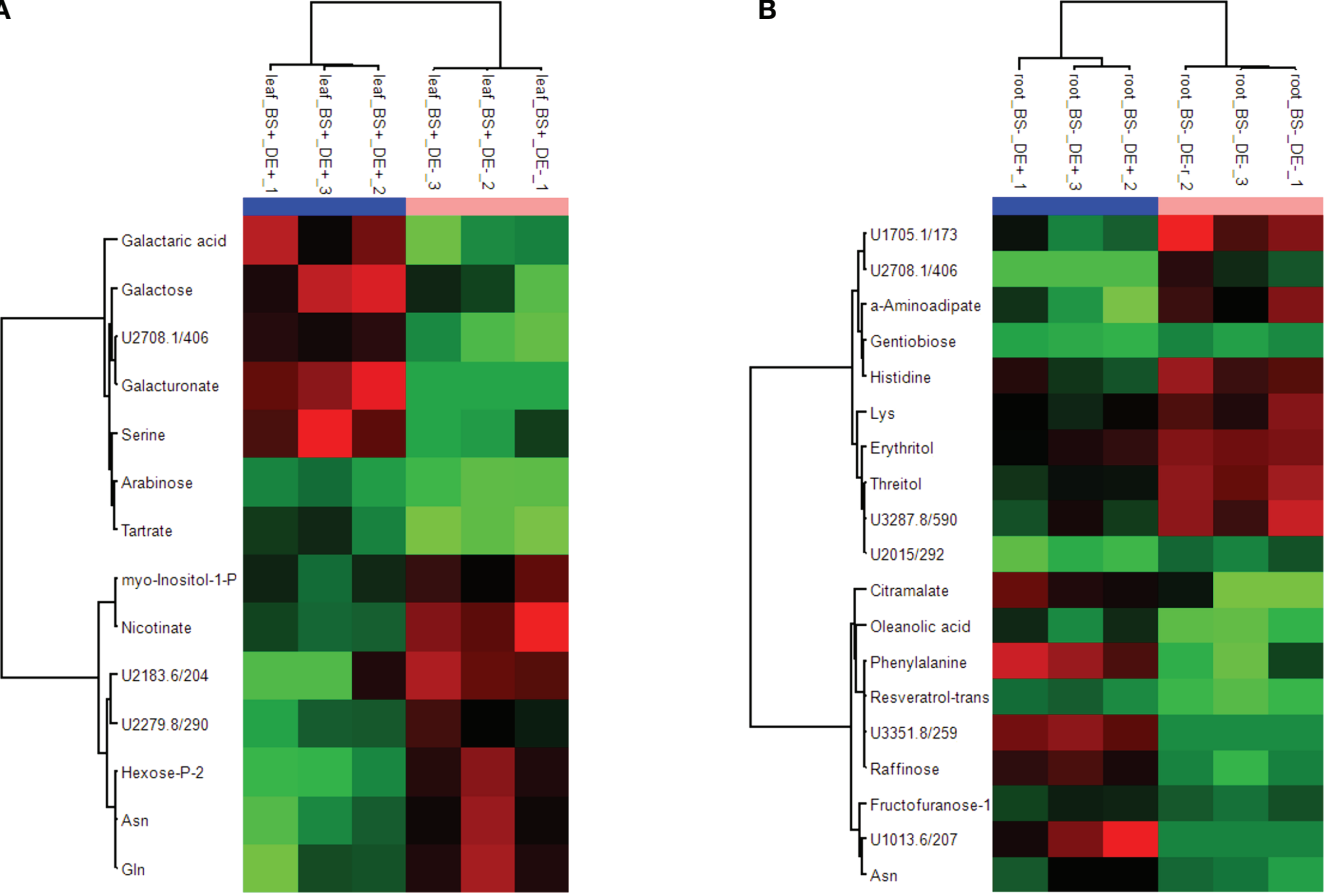
Heatmap showing metabolites differently accumulated in leaves and roots of plantlets treated by BS and/or DE. Grapevine micro-cuttings were transplanted in a solid medium supplemented with BS 0.1% (v/v, “BS+”) or water (“BS-”, as control). At 4 wpt, they were divided into two sets: one treated by immersion in a defense elicitor solution (DE+) and the other one in water as control (DE-). Two days later, leaves, stems and roots were collected. Extracts were then prepared and analyzed by GC-MS. Significant differences were observed for BS+ modality in leaves **(A)**, and BS- modality in roots **(B)**. Data were processed using ANOVA *(P <* 0.05).

Whether the DE had different effects on the metabolism of BS+ and BS- plantlets was investigated. Note that no phytotoxicity was observed following DE treatment. At 4 wpt, BS- and BS+ grapevines were divided into two sets, with one treated with the defense elicitor (“DE+”) and the other with ultrapure water (“DE-”) as the control. Root, stem, and leaf samples were collected 2 days post DE or water treatment, and metabolites were analyzed by GC–MS. Hierarchical clustering analysis of all data obtained (BS+ and BS- samples collected at 4 wpt; BS+/DE+, BS+/DE-, BS-/DE+, and BS-/DE- samples collected at 4 wpt + 2 d) clearly separated the three types of organs (data not shown). A specific HCA was then performed for each set of organ samples. The BS+ and BS- samples collected at 4 wpt formed a separate subgroup for stem and leaves but not for roots. For root metabolites, all BS+ samples were separated from BS- ones, independently of sampling time and DE treatment. There was no discrimination between DE+ and DE- samples for stems. Notably, DE+ and DE- samples were separated only for BS+ leaf samples and BS- root samples (**Supplementary Figure S5**). More precisely, there were higher amounts of galactaric acid, galactose, galacturonate, and serine and lower amounts of myo-inositol-1-P, nicotinate, a phosphorylated hexose, asparagine, and glutamine in BS+/DE+ leaves than in BS+/DE- ones (**Figure 6A**). There were higher amounts of citramalate, oleanolic acid, phenylalanine, *t*-resveratrol, raffinose, fructofuranose, and asparagine and lower amounts of α-aminoadipate, gentiobiose, histidine, lysine, erythritol, and threitol in BS-/DE+ leaves than in BS-/DE- ones (**Figure 6B**).

**Figure 7:**
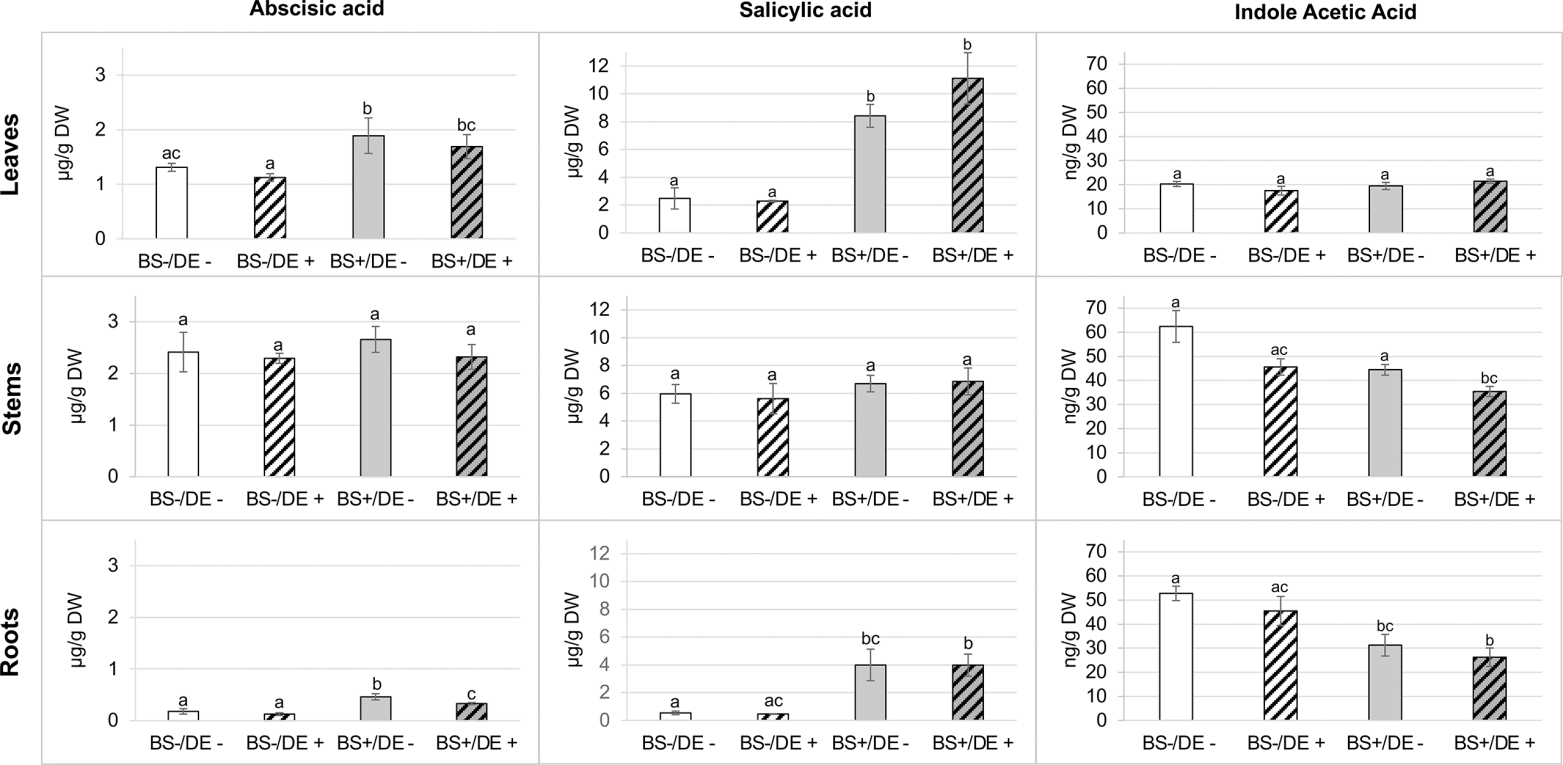
Effect of BS treatment on abscisic acid, (ABA), salicylic acid (SA) and indole acetic acid (IAA) content of grapevine plantlet leaves, stems and roots. Grapevine micro-cuttings were transplanted in a solid medium supplemented with BS 0.1% (v/v, “BS+”) or water (“BS-” as control). At 4 wpt, they were divided into two sets: one treated by immersion in a defense elicitor solution (DE+) and the other one in water as control (DE-). Two days later, leaves, stems and roots were collected. Extracts were prepared and analyzed by LC-MS. The bars represent the standard deviations and a significant difference is observed when the treatments have no letter (a or b) in common (*P* < 0.05) after a Kruskall-Wallis test.

For phytohormones (**Figure 7**), ABA and SA were found in similar concentrations in stems of the four sample types (2.29 to 2.65 µg/g DW and 5.61 to 6.85 µg/g DW, respectively). Concentrations of ABA and SA were higher in leaves than in roots. Amounts of ABA were significantly higher in leaves and roots of BS+/DE- and BS+/DE+ plantlets than in BS- ones. For BS+ samples, ABA levels were similar in leaves and were significantly lower in DE+ root samples than in DE- ones. The DE significantly reduced ABA concentration, but only in roots of BS+ plantlets. In leaves and roots, the concentration of SA was significantly higher in BS+ plantlets than in BS- ones, but there was no difference between BS+/DE- and BS+/DE+ samples. The DE did not affect SA concentration, regardless of the organ considered. For IAA, higher amounts were found in stems and roots than in leaves. In leaves, concentrations were similar for all treatments. The DE treatment significantly reduced IAA concentration in stems, but only in BS+ plantlets.

Composition of the DE was also determined (**Supplementary File 1**). In the GC–MS analysis, 35 compounds were detected, including “unknown” metabolites (14%), organic acids including galacturonic acid (43%), sugars (31%), and others (9%), and phosphorylated compounds (3%) (data not shown). Neither IAA nor ABA were detected, but SA was detected at a mean concentration of 280 ng/g DW.

### The biostimulant BS “primes” elicitor-induced accumulation of phenolic compounds and expression of defense genes in leaves

**Figure 8:**
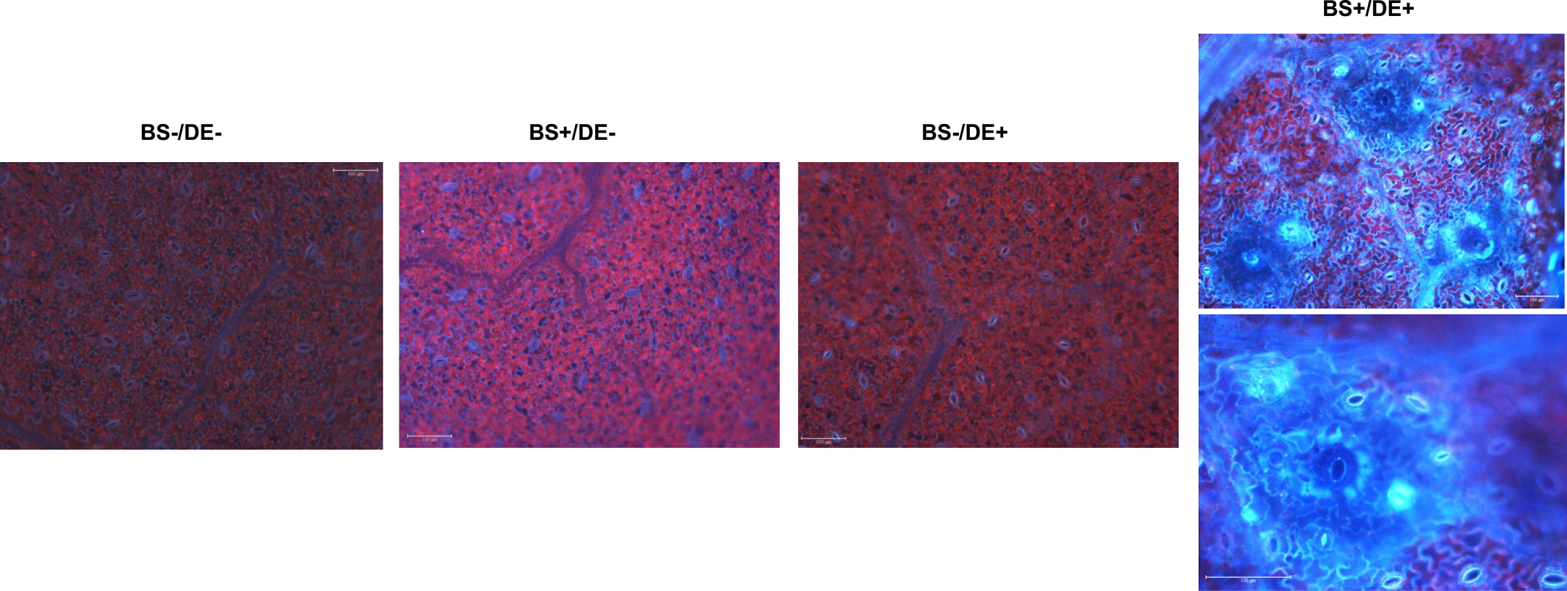
Observation of grapevine leaves by epifluorescence microscopy. Micro-cuttings were transplanted in a solid medium supplemented by BS 0.1% (v/v, “BS+”) or water (“BS-”, as control). At 4 wpt, they were divided into two sets: one treated by immersion in a defense elicitor solution (DE+) and the other one in water as control (DE-). Two days later, leaves were detached from plantlets and their lower side was observed by fluorescence microscopy. Bars represents 100 μm.

The accumulation of phenolic compounds in leaves was evaluated using fluorescence microscopy at 2 days post DE treatment because some compounds fluoresce in blue purple under a specific wavelength. No fluorescence was observed in BS-/DE-, BS+/DE-, or BS-/DE+ leaves, except for naturally low levels at stomatal sites (**Figure 8**). By contrast, strong fluorescence was observed primarily in veins, cell walls, and stomata in leaves of BS+/DE+ plantlets (**Figure 8**).

**Figure 9:**
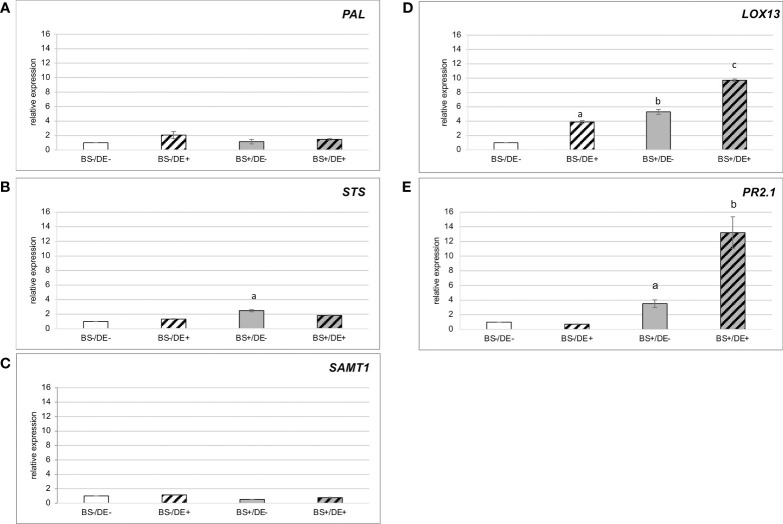
Relative expression of defense-related genes in leaves of plantlets treated by BS and/or DE. Grapevine micro-cuttings were transplanted in a solid medium supplemented with BS 0.1% (v/v, “BS+”) or water (“BS-”, as control). At 4 wpt, they were divided into two sets: one treated by immersion in a defense elicitor solution (DE+) and the other one in water as control (DE-). Two days later, leaves were collected. The expression of five genes encoding a **(A)** phenylalanine ammonia-lyase 1 (PAL), **(B)** stilbene synthase (STS), **(C)** salicylic acid methyltransferase (SAMT1), **(D)** lipoxygenase 13 (LOX13), **(E)** β-1.3 glucanase (PR2.1) were investigated by qRT-PCR. Results represent relative fold expression calculated with the 2^-ΔΔCT^ method and normalized against the two reference genes *EF1α* and *VATP16* as internal controls for each respective time point. Data represent mean of three technical replicates of one biological repetition. The bars represent the standard deviations and a significant difference is observed when the treatments have no letter (a or b) in common (*P* < 0.05; Kruskall-Wallis test).

Expression of a set of genes that are induced in grapevine by defense elicitors was studied in samples collected at 2 days post DE treatment (**Figure 9**). Expression of *PAL* and *SAMT1* was not induced (**Figures 9A–C**), whereas expression of *STS* was slightly induced in BS+/DE- samples (**Figure 9B**). Expression of *LOX13* was significantly induced by BS and DE treatments alone, but the highest expression was in the DE treatment of biostimulated plantlets (BS+/DE+) (**Figure 9D**). Expression of *PR2* was not induced by DE treatment alone but was slightly induced in BS+/DE- plantlets and most induced in BS+/DE+ ones (**Figure 9E**).

### The biostimulant BS conditions defense-elicitor induction of grapevine resistance to downy mildew

**Figure 10:**
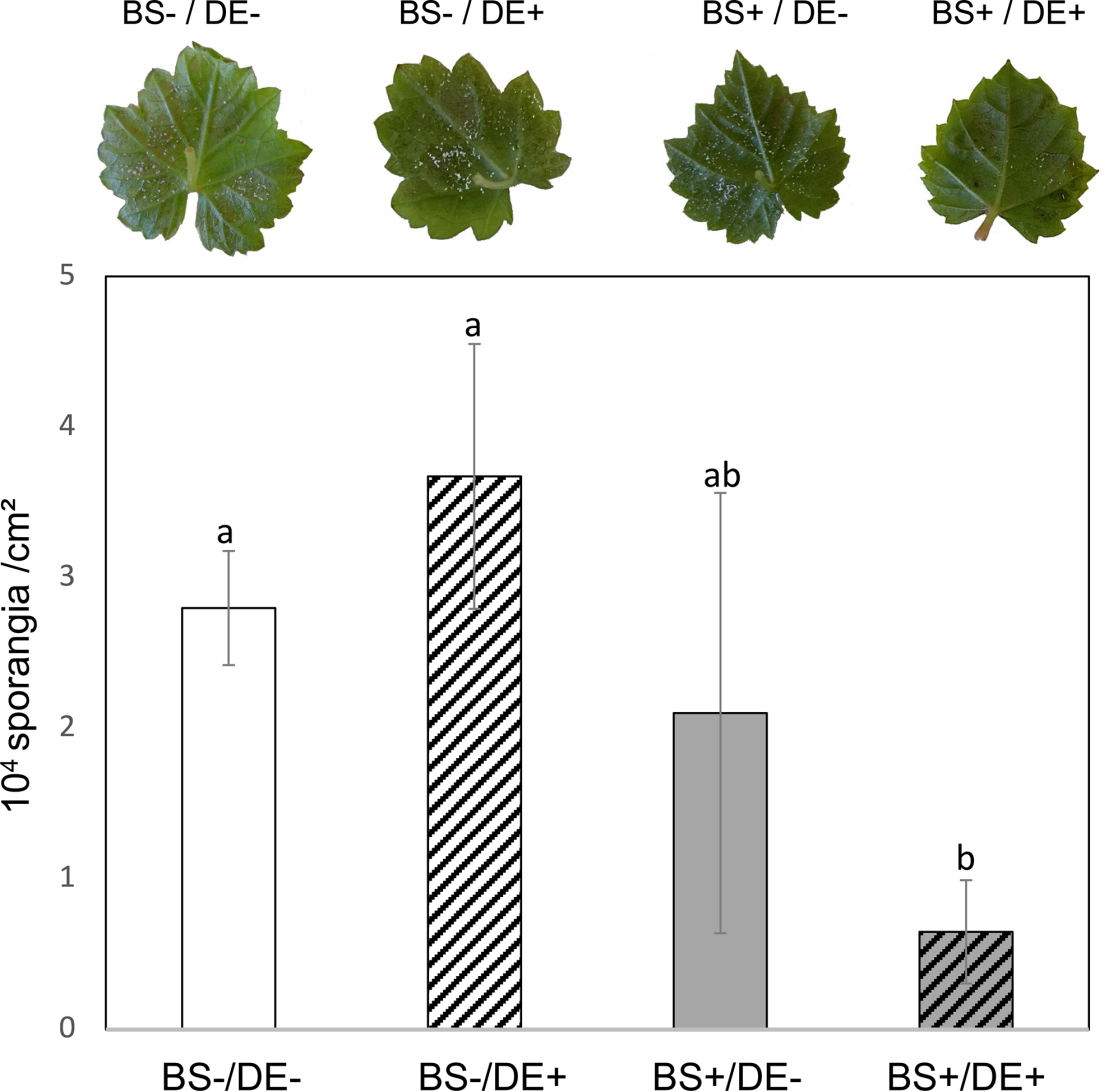
Evaluation of *Plasmopara viticola* sporulation according to the different treatments. Grapevine micro-cuttings were transplanted in a solid medium supplemented with BS 0.1% (v/v, “BS+”) or water (“BS-”, as control). At 4 wpt, they were divided into two sets: one treated by immersion in a defense elicitor solution (DE+) and the other one in water as control (DE-). Two days later, leaves were detached and inoculated with a *P. viticola* sporangia suspension. Sporulation was evaluated at 7 days post inoculation. Top: Photographs representative of the pathogen sporulation observed on the lower surface of the leaves. Bottom: histogram showing the evaluation of the density of sporangia per leaf area (cm^2^). The bars represent the standard deviations and a significant difference is observed when the treatments have no letter (a or b) in common (*P*<0.05; Kruskall-Wallis test).

Last, whether biostimulation improved DE-induced resistance of grapevine against downy mildew was investigated. At 2 days post DE treatment, leaves were inoculated with *P. viticola*, and sporulation of the pathogen was assessed at 7 days post inoculation (dpi). Sporulation was sparse (**Figure 10**, photographs) and did not allow reliable and representative image data to be acquired for analysis. However, frequency and intensity of sporulation of the pathogen were considerably lower in BS+/DE+ plantlet leaves, compared to other treatments (**Figure 10**, right photograph). To more accurately assess the effect of different treatments on pathogen sporulation, sporangia density per leaf area (cm^2^) was estimated. Notably, DE did not induce protection of leaves in BS- plantlets. Only the BS+/DE+ treatment led to a significant reduction in *P. viticola* sporulation and therefore impaired the production of secondary inoculum. The density of sporangia per leaf unit area decreased by more than 25%, with 0.65 sporangia/cm^2^ in BS+/DE+ plantlets compared with 2.79 sporangia/cm^2^ in BS-/DE- plantlets (**Figure 10**).

**Figure 11:**
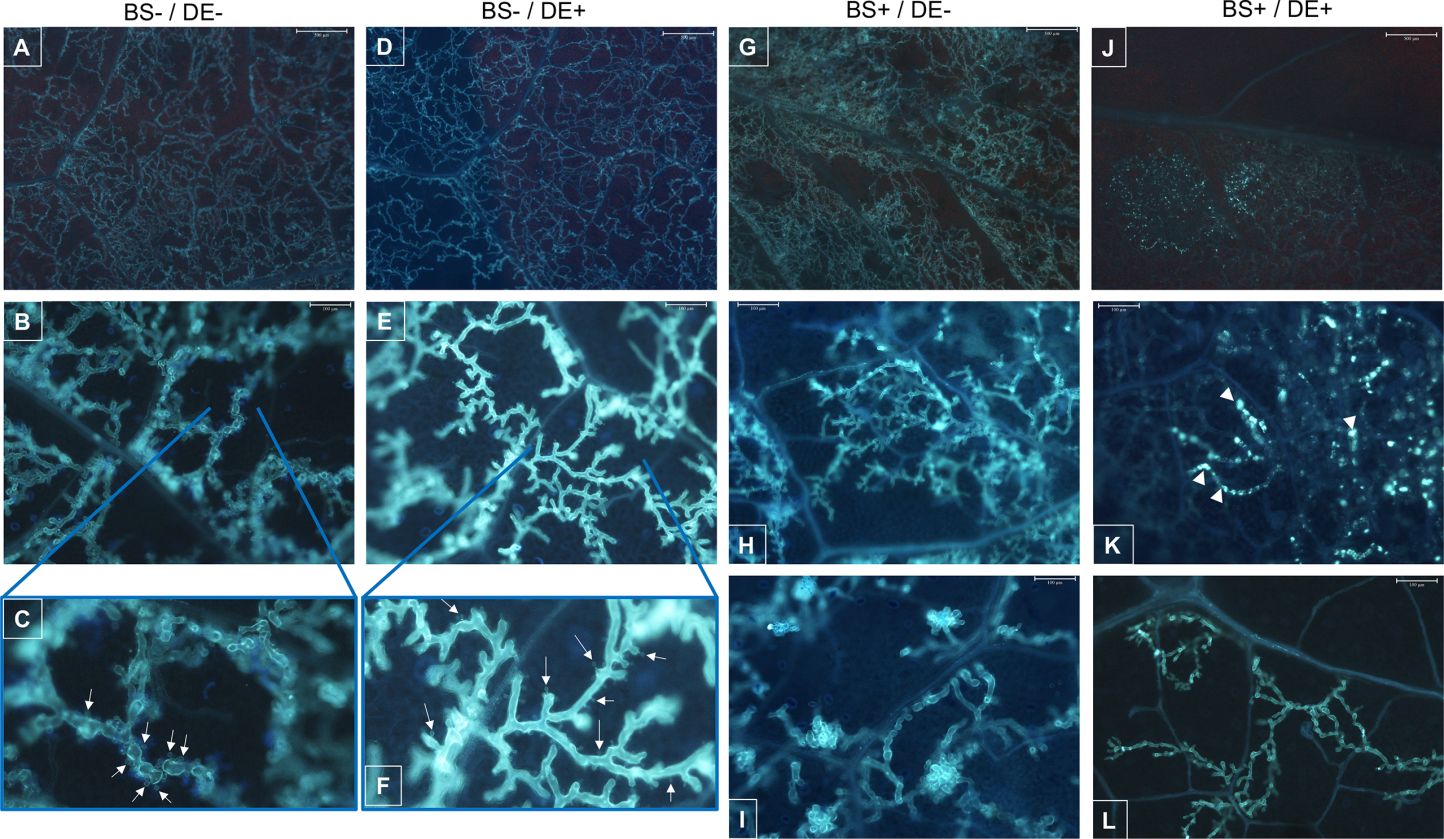
Cytological observations of the mycelial colonization of grapevine leaves by *Plasmopara viticola*. Micro-cuttings were transplanted in a solid medium supplemented with BS 0.1% (v/v, “BS+”) or water (“BS-”, as control). At 4 wpt, they were divided into two sets: one treated by immersion in a defense elicitor solution (DE+) and the other one in water as control (DE-). Two days later, leaves were detached and inoculated with a *P. viticola* sporangia suspension. The mycelial development into leaves was observed at 7 dpi, by microscopy, under UV, after aniline blue staining. **(A–C)** samples collected from BS-/DE- plantlets; showing the importance of mycelial colonization **(A)**, the typical structure of hyphae and their branching **(B)** as well as the presence of haustoria (**C**, arrows). **(D–F)** samples collected from BS-/DE+ plantlets; showing the importance of mycelial colonization **(D)**, the structure of hyphae and their branching **(E)** as well as the presence of haustoria (**F**, arrows). **(G–I)**: samples collected from BS+/DE- plantlets; showing the importance of mycelial colonization **(G)**, the structure of hyphae and their branching **(H, I)**. **(J–L)**: samples collected from BS+/DE+ plantlets; showing a reduced internal colonization **(J)**, with, in some areas, altered hyphae presenting inside a material with affinity for aniline blue, therefore composed of β-1,3 glucan (**J; K**, arrowheads). In other areas, hyphae appear less affected but are sparse and haustoria are not apparent **(L)**. The scale bar corresponds to 500 μm **(A, D, G, J)**, or 100 μm **(B, E, H, I, K, L)**.

The internal colonization of leaves by *P. viticola* was evaluated by using a cytological approach (**Figure 11**). In control plants (BS-/DE-), there were many branched hyphae with typical structure (**Figures 11A, B**) and many haustoria (**Figure 7C**, arrows). A similar profile was observed in BS-/DE+ plantlets (**Figures 11D–F**), suggesting that DE alone did not significantly affect pathogen development. The BS alone (BS+/DE-) did not limit pathogen internal colonization, which remained dense (**Figure 11G**) but haustoria were rare (**Figures 11H, I**), in contrast to BS-/DE- plantlets. In leaves of BS+/DE+ plantlets, internal colonization was reduced considerably (**Figure 11J**), and very shiny hyphae were observed in some areas (**Figures 11J, K**). In those areas and after aniline blue staining, UV-fluorescent clumps were frequently observed inside the hyphae (**Figure 11K**, arrowheads). Furthermore, in areas where the hyphae were sparse and less affected, haustoria were not easily observed (**Figure 11L**), in contrast to the control (**Figure 11C**) and DE treatment alone (**Figure 11F**).

## Discussion

The objective of this study was to assess whether biostimulation could improve the efficiency of elicitor-induced resistance of grapevine leaves to downy mildew. The BS used was a plant extract with added iron sulfate heptahydrate and zinc sulfate monohydrate. In previous experiments, the BS had no antifungal properties (Laboratoires Goëmar, pers. com.).

An original system was developed using *in vitro* grapevine plantlets that relatively quickly assessed BS effects. The system is the first to use such plant material to test the effect of a biostimulant on grapevine. Similar experiments are generally performed in greenhouses or the field and are also long-lasting and can be influenced by environmental factors such as soil and climatic conditions, as well as cultural practices. Despite limitations (e.g., BS application only in the culture medium, absence of reproductive organs, not adapted for microbial biostimulants), such *in vitro* model is used in controlled environmental conditions and uses a culture medium of known composition. Moreover, with this model, the complete root system can be observed. Therefore, this model is adapted for lab screening of biostimulants active in grapevine and to study effects on plantlets. Rayorath et al. (2008) also used *in vitro* assays to evaluate the promoting activity of *A. nodosum* on Arabidopsis growth. It is indeed useful to develop phenotyping for screening and study of the mode of action of biostimulants even if assays remain essential in the realistic field conditions for use in agriculture (Rouphael et al., 2018). In the system used in this study, the BS was added to the culture medium and therefore applied to roots. Previous studies conducted with potted grapevines in greenhouses (Mugnai et al., 2008; Meggio et al., 2020) or in the field (Arioli et al., 2021; Irani et al., 2021) reported positive effects of biostimulants provided at the root level.

For the three concentrations of the BS (0.03%, 0.1%, and 0.3% v/v), a dose-dependent effect was observed on development of root and shoot systems. At the highest concentration, phytotoxicity was observed. The intermediate concentration of 0.1% BS induced faster and more homogeneous plantlet development (bud opening and date of “first expanded leaf”) than the other concentrations. Effects of biostimulants on grapevine development have been previously reported, although such studies are limited compared with those on yields and wine quality (for review, see Yilmaz and Gazioglu Şensoy, 2021; Cataldo et al., 2022; Monteiro et al., 2022; Samuels et al., 2022). For example, Mugnai et al. (2008) reported that marine bioactive substances had beneficial effects on the development of potted, grafted grapevines (*V. vinifera* ‘Sangiovese’ grafted on 420A rootstock), and improved ammonium and/or potassium absorption. In winter oilseed rape, Billard et al. (2014) observed that an extract of *A. nodosum* and humic acid stimulated root growth and macronutrient uptake. Whether the BS also stimulated nutrient uptake would be of interest and could be determined by analyzing mineral contents of roots and leaves. Moreover, combinations of biostimulants with nutrient elements can increase effects compared with biostimulants alone. For grapevine in the field, El-Boray et al. (2015) reported increases in effects of fulvic acid when combined with iron sulfate, zinc sulfate, and manganese sulfate. Mostafa et al. (2017) found increases in effects when fulvic acid was combined with magnesium sulfate and potassium sulfate. Among the parameters considered in those studies were bud burst, shoot length, and leaf surface. Because the BS contained iron sulfate and zinc sulfate, those nutrients might have increased the effects observed. The effects of the BS observed in this study (faster and more homogeneous plantlet development) should be investigated further in nurseries and vineyards. If effects are still observed, the BS could be exploited for practical applications, including vine production in nurseries, vine planting, replacing dead vines in vineyards, and treatment after frost or hail. However, two phenotypes were observed for some BS+ plantlets that had altered shoot and root development. Such secondary effects should also be investigated further in order to understand the origin and how to avoid such problems.

The positive effects of the BS on shoot development were not observed from 4 wpt. Whether the positive effects stopped because of decreases in amounts of active BS components in the culture medium (suggesting that additional applications would be needed) or long-term negative effects of BS could not be determined. Moreover, the phenotype of some plantlets was altered, and decreases in number of lateral roots likely reduced capacity of those plantlets for nutrient absorption, with possible effects on development and absorption of active BS components. To use in practical applications, it needs to be determined whether foliar applications produce the positive effects of the BS on plant development without the negative effects, because foliar application would be easier to perform in a vineyard than soil application.

The BS in this study had different effects on metabolomes and hormone contents of different plantlet organs. Metabolite analysis in this study focused primarily on primary metabolites, because there were only a few secondary metabolites (phenolic compounds) annotated in the data bases used. The metabolomes of BS+ plantlet stems and leaves and particularly roots were strongly affected. Compared with BS- plantlets, the metabolites with greater accumulations in BS+ plantlets were mainly amino acids and sugars in leaves, amino acids and organic acids in stems, and organic acids and sugars in roots. Thus, when applied to roots, the BS affected plant carbon and nitrogen metabolism. However, whether the BS increased metabolite synthesis or decreased metabolism of metabolites could not be determined. In previous studies on biostimulant treatment of plant organs, the focus was generally on metabolomes of fruits, and few focus on leaf or root metabolomes. Ertani et al. (2014) reported on changes in metabolites of leaves of *Capsicum chinensis* treated by two plant-derived biostimulants. Paul et al. (2019a); Lucini et al. (2020), and Ceccarelli et al. (2021) reported on changes in metabolomes of tomato roots and/or leaves following application of plant-derived protein hydrolysates. Bodin et al. (2020) reported on time-dependent changes in flavonol and anthocyanin contents in grapevine leaves treated by a seeweed extract enriched with oligo-elements. Because the BS was provided in the culture medium in this study, whether the metabolites that accumulated in roots of BS+ plantlets were those contained in the BS was examined. Among the 56 compounds with increased accumulation in BS+ plantlet roots, only nine were BS components. Moreover, the distribution of biochemical categories in the BS and roots was highly different. Thus, it was concluded that the changes in the root metabolite profile were due to effects of the BS on plantlet metabolism rather than accumulation of absorbed BS components. This conclusion could be extended to the changes observed in leaves and stems. The BS also affected concentrations of ABA, SA, and IAA. Paul et al. (2019a); Paul et al. (2019b) also reported hormonal regulation in tomato plants treated by a plant-derived protein hydrolysate, with decreases in cytokinin and gibberellin amounts and an increase in salicylate amount. Lucini et al. (2020) reported positive effects of a protein hydrolysate on tomato root length, and those effects were associated with changes in phytohormones and secondary metabolite levels, suggesting auxin-like activity. Bodin et al. (2020) observed a decrease in the SA content of leaves treated by a seaweed extract. Wally et al. (2013) showed that application of a *A. nodosum* extract to *in vitro* grown *Arabidopsis* plants lead to alteration of the root phenotype, as a result of the modulation of the biosynthesis, quantity and ratio of cytokinins, auxins, and ABA metabolites. The authors demonstrated that the increases were due to phytohormone pathway regulation and not to hormone content of the extract. When hormone concentrations determined in the BS and samples were compared in this study, the same conclusion was reached.

The effect of BS treatment on the efficiency of elicitor-induced resistance against grapevine downy mildew was also assessed. The DE was applied on 4-week-old plantlets, which was the time beyond which the BS no longer had a positive effect on plantlet growth. The DE contains oligopectins which are known to stimulate plant defenses (Ridley et al., 2001). The DE was previously studied using grapevine cell suspensions and plants grown in greenhouses (Krzyzaniak et al., 2018). Notably, in this study, the DE did not induce expression of defense genes or defense compounds (as observed by fluorescence) or provide protection in BS- plantlets. Those results remain unexplained. It is possible that responses to elicitors are different between *in vitro* plantlets and plants grown in greenhouses. However, such differences may be specific to DEs, because previous studies reported similar responses following defense activation by UV-light or aluminum chloride (Borie et al., 2003). Differences may also be due to application of the DE by immersion, instead of foliar spray. However, active defense stimulation by immersion has been reported (Varnier et al., 2009). Notably, the results in this study suggested that the BS acted by priming the DE effect, because expression of two defense genes was induced and defense compounds strongly accumulated only in BS+/DE+ leaves. These changes were correlated with induced resistance against downy mildew. This report is the first of such effects.

The induced defenses and resistance to downy mildew could be due to BS-induced SA accumulation, because the highest concentrations were detected in roots and leaves of BS+ plantlets. Although SA was detected among the BS components, the concentrations found in the different organs indicated that the BS clearly induced SA accumulation. The results also suggested systemic acquired resistance was induced. Agarwal et al. (2016) reported a significant increase in SA concentration in *Lycopersicon esculentum* leaves treated by K-sap (sap of the seaweed *Kappaphycus alvarezii*). In contrast to results in the current study, contents of hormones ABA and IAA, as well as zeatin, also increased and defense genes were induced. Therefore, the K-sap may act as both defense elicitor and biostimulant in that study. Although definition and regulation distinguish plant biostimulants and defense elicitors, some biostimulants can activate plant defenses (Bodin et al., 2020, for example in grapevine). In this study, genes involved in the phenylpropanoid pathway (*PAL* and *STS*) were not induced at 2 days post DE treatment. Because phenolic compounds increased (autofluorescence observations), induction might have occurred earlier. A complete kinetic study of gene expression and hormone levels should be performed in order to better understand gene regulation and possible hormonal crosstalk in the experimental conditions of this study. Upregulation of the expression of *PR2.1* encoding a glucanase could explain, at less in part, the reduction in *P. viticola* colonization, because β-glucans are the main component of oomycete cell walls.

The BS might also affect *P. viticola.* Although hyphal development was not limited in BS+ leaves, haustoria were not easily observed. Moreover, in leaves of BS+/DE+ plantlets, hyphae were scarce, with some that were very shiny with frequent fluorescent clumps inside. Because aniline blue dye is specific for β-1,3 glucans, that observation, which indicated an alteration in parasite behavior, suggested an accumulation of callose or laminarin (i.e., storage sugar for the oomycete) in response to treatment. In areas where the hyphae were sparse and less affected, it was also not easy to observe haustoria.

Thus, the mode of action of the BS tested in this study is complex. As reviewed by De Saeger et al. (2020) for *A. nodosum* extracts, positive effects of biostimulants on plants can be due to various compounds and result from regulation of different pathways, making it difficult to elucidate modes of action. The results obtained in this study require confirmation in the field. Preliminary experiments in the field have been conducted but did not demonstrate any differences between BS+/DE+ and DE+ alone. However, BS and DE were applied in combination. For experiments in controlled conditions in the current study, BS was applied before DE so that it had enough time to affect the plantlet. Therefore, experiments in the field should be repeated with BS treatments applied before defense elicitors application and with sufficient time (to be determined) for a BS to condition plants.

## Conclusion

This study showed that grapevine *in vitro* plantlets can be used to study biostimulants. This model allowed the characterization of the effects of a BS on the development of both plant aerial and root systems, and on the metabolomes and phytohormone contents of leaves, roots, and stems. It also highlighted that the biostimulant used primed elicitor-induced defenses and resistance against downy mildew. This work thus demonstrates, for the first time, that biostimulation might be a lever to increase the efficiency of plant defense elicitors. It also open the way for new studies to verify if such effects also occur in the complexity and diversity of vineyard conditions and for other crops.

## Data availability statement

The raw data supporting the conclusions of this article will be made available by the authors, without undue reservation.

## Author contributions

LJ, ST, and YK performed experiments (plantlets culture, development monitoring, sampling). LJ and ST performed phenotyping, analysis of gene expression, induced resistance assays and visualization of mycelial colonization. LJ and CL-G performed statistical analysis. GC performed sample preparation for GC-MS analysis, GC-MS analysis, and data process. SC performed sample preparation for LC-MS analysis, LC-MS analysis, and data process. GM supervised GC-MS and LC-MS analysis. EM provided BS, DE, and recommendations for their use. CL-G, LJ, MA, M-CH, and ST contributed to experimental design and data interpretation. LJ, ST, and CL-G contributed to draft the manuscript. MA drafted led the project. All authors contributed to the article and approved the submitted version.

## Funding

This work was supported by Région Bourgogne Franche-Comté, European Union Program (Feder), BPI France and Bureau Interprofessionnel des Vins de Bourgogne (FUI Iris+ and PARI Vigne Vin programs), Goëmar Laboratories.

## Acknowledgments

This work has benefited from the support of IJPB’s Plant Observatory technological platforms. We thank G. Lecollinet (Laboratoires Goëmar) for his help in finalizing this paper. We also thank JM Joubert who allowed the initiation of this project.

## Conflict of interest

The authors declare that the research was conducted in the absence of any commercial or financial relationships that could be construed as a potential conflict of interest.

## Publisher’s note

All claims expressed in this article are solely those of the authors and do not necessarily represent those of their affiliated organizations, or those of the publisher, the editors and the reviewers. Any product that may be evaluated in this article, or claim that may be made by its manufacturer, is not guaranteed or endorsed by the publisher.

## Supplementary material

Supplementary Table 1 Sequences of the primers used for qRT-PCR analyses.

Supplementary File 1 List of metabolites contained in BS and DE. Metabolites identified in BS and DE by GC-MS are listed by alphabetical order.

Supplementary Figure 1 Outline of the experiments. For this study, one hundred and sixty-eight plantlets were used per condition (BS+/BS-). At 4 wpt, 120 plantlets were treated either with a defense elicitor (DE+) or water (DE-) as control (60 per condition) and 48 ones were used for analyses.

Supplementary Figure 2 Effect of the biostimulant BS on the beginning of development of *in vitro* grapevine plantlets. Micro-cuttings were transplanted in a solid medium supplemented with BS (0.1% v/v) or water (as control), and the date of the “first expanded leaf” stage was compared. **(A)**: time (mean number of days) between the date of the micro-cutting transplantation and the date of the first expanded leaf stage. **(B)**: cumulative percentage of plantlets that have reached the one expanded leaf stage. Asterisks indicate significant differences with Student’s t-test with ****P* < 0.001.

Supplementary Figure 3 Heatmap showing the detected metabolites differently accumulated in leaves, stems and roots of plantlets following treatment by a biostimulant. Grapevine micro-cuttings were transplanted in a solid culture medium supplemented with BS 0.1% (v/v, “BS+”) or water (“BS-” as control). At 4 wpt, plantlets were collected and dissected to fdistinct root, leaf and stem samples. Methanolic extracts were prepared and analyzed by GC-MS. Data were processed using ANOVA (*P* < 0.05); distance measure used is Pearson’s correlation, and clustering algorithm is average. In red: metabolites more accumulated in BS+ than in BS- organs.

Supplementary Figure 4 List of the metabolites differently accumulated in leaves, stems and roots of BS-treated plantlets, compared to control ones. Grapevine microcuttings were transplanted in a solid culture medium supplemented with BS 0.1% (v/v, “BS+”) or water (“BS-”, as control). At 4 wpt, plantlets were collected and dissected to distinct root, leaf and stem samples. Methanolic extracts were prepared and analyzed by GC-MS. Data were processed using ANOVA (*P* < 0.05). Red : metabolites more accumulated in BS+ than in BS- organs, In green: metabolites less accumulated in BS+ than in BS- organs.

Supplementary Figure 5 Heatmap showing the metabolites differently accumulated in leaves, stems and roots of plantlets following treated by BS or DE. Grapevine micro-cuttings were transplanted in a solid medium supplemented with BS 0.1% (v/v, “BS+”) or water (“BS-”, as control). At 4 wpt, they were divided into two sets: one treated by immersion in a defense elicitor solution (DE+) and the other one in water as control (DE-). Two days later, plantlets were collected and dissected to distinct leaf, stem and root samples. Methanolic extracts were prepared and analyzed by GC-MS. Data were processed using ANOVA (*P* < 0.05); distance measure used is Pearson’s correlation, and clustering algorithm is average.

## Abbreviations

BS, Biostimulant; DE, Plant Defense Elicitor.
