# Supplementary material for: Biostimulation can prime elicitor induced resistance of grapevine leaves to downy mildew
Source: Front Plant Sci. 2022 Nov 9;13:998273. doi: 10.3389/fpls.2022.998273 (PMC9682252; doi:10.3389/fpls.2022.998273)
Supplement: Supplementary Table 1 — Sequences of the primers used for qRT-PCR analyses. [file Table_1.pdf]

			Gene symbol	Accession N°	Forward Primer (5'->3')	Reverse Primer (5'->3')	Reference
Reference genes	EF1-α elongation factor		*EF1α*	XM_002284888	GAACTGGGTGCTTGATAGGC	AACCAAAATATCCGGAGTAAAAGA	Dubreuil-Maurizi et al 2010
	V-ATPase 16 kDa proteolipid subunit 5		*VATP16*	XM_002269086.1	CTTCTCCTGTATGGGAGCTG	CCATAACAACTGGTACAATCGAC	Gamm et al 2011
Defense-related genes	Phenylpropanoid pathway	Phenylalanine ammonia lyase	*PAL*	XM_002268220	AGTCTCCATGGACAACACCCG	TGCTCAGCACTTTCGACATGG	Dubreuil-Maurizi et al 2010; Aziz et al 2003
		Stilbene synthase	*STS*	X76892.1	AGGAAGCAGCATTGAAGGCTC	TGCACCAGGCATTTCTACACC	Trouvelot et al 2008
	Signaling	SA Methyl Transferase	*SAMT1*	XM_002262982.1	GGGCTGGAGAACAAAGGGAA	CGACCGACACCTGAGAAACA	this study
	Oxylipins	Lipoxygenase-13	*LOX13*	XM_002285538.1	CATGGGTTGCTTCCAAGTTT	CAGGACTGCTGTCTGGATCA	Krzyzaniak et al 2018
	PR proteins (spe oomycetes)	Beta-1,3-glucanase	*PR2.1*	XM_002277133.1	ATGCTGGGTGTCCCAAACTCG	CAGAACAAACTGCGCAAACCGT	Dubreuil-Maurizi et al 2010; Aziz et al 2003
