# Supplementary figures and images for: Biostimulation can prime elicitor induced resistance of grapevine leaves to downy mildew
Source: Front Plant Sci. 2022 Nov 9;13:998273. doi: 10.3389/fpls.2022.998273 (PMC9682252; doi:10.3389/fpls.2022.998273)

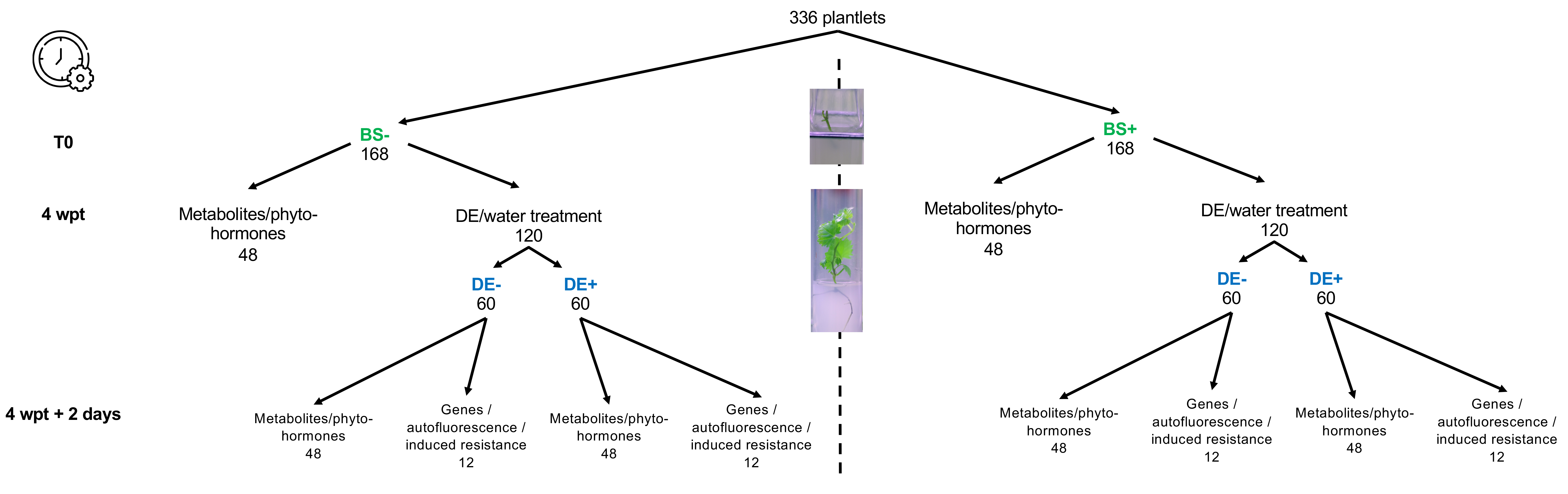

Supplement: Supplementary Figure 1 — Outline of the experiments. For this study, one hundred and sixty-eight plantlets were used per condition (BS+/BS-). At 4 wpt, 120 plantlets were treated either with a defense elicitor (DE+) or water (DE-) as control (60 per condition) and 48 ones were used for analyses. [file Image_1.tif]

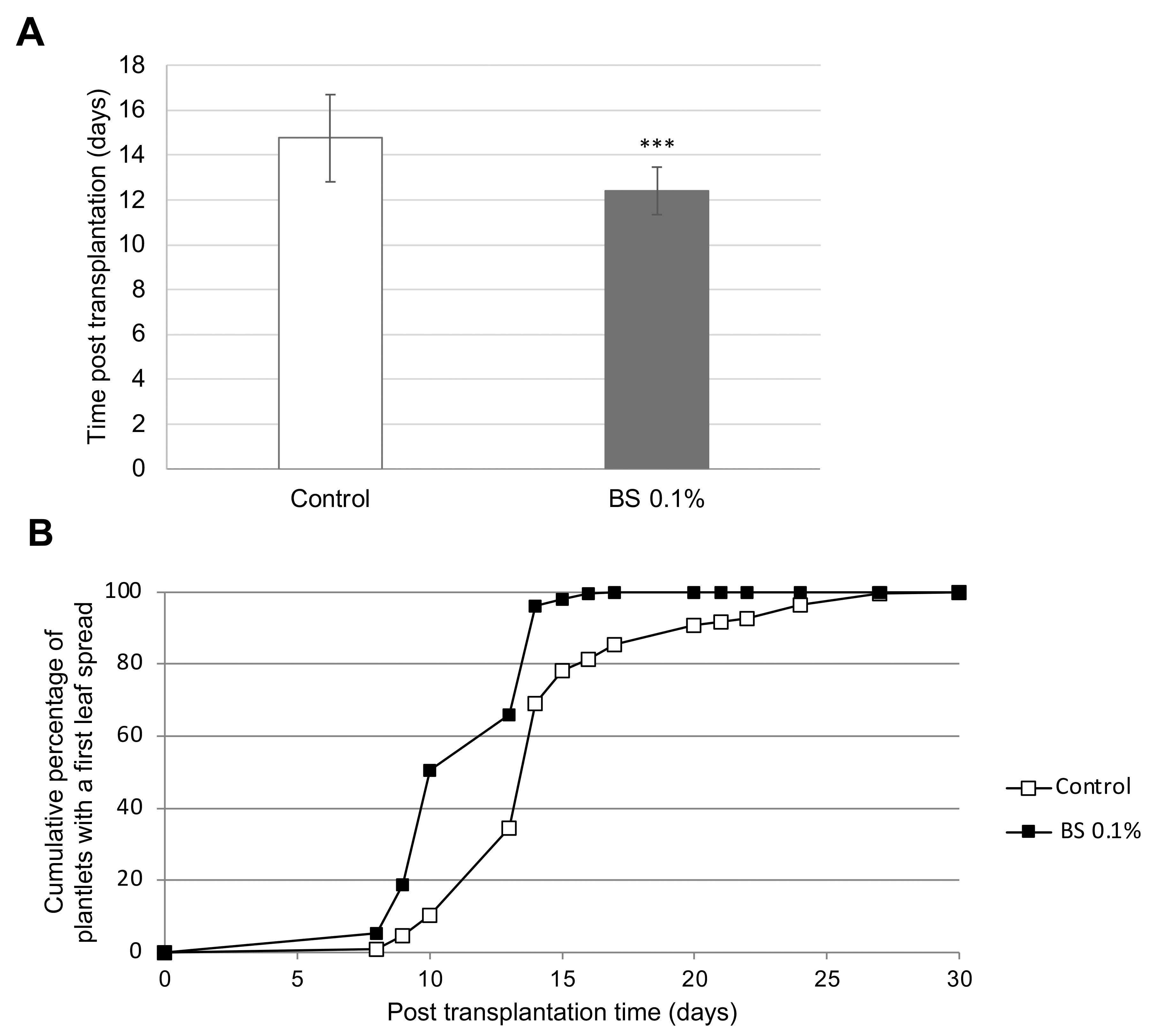

Supplement: Supplementary Figure 2 — Effect of the biostimulant BS on the beginning of development of in vitro grapevine plantlets. Micro-cuttings were transplanted in a solid medium supplemented with BS (0.1% v/v) or water (as control), and the date of the “first expanded leaf” stage was compared. (A): time (mean number of days) between the date of the micro-cutting transplantation and the date of the first expanded leaf stage. (B): cumulative percentage of plantlets that have reached the one expanded leaf stage. Asterisks indicate significant differences with Student’s t-test with ***P < 0.001. [file Image_2.tif]

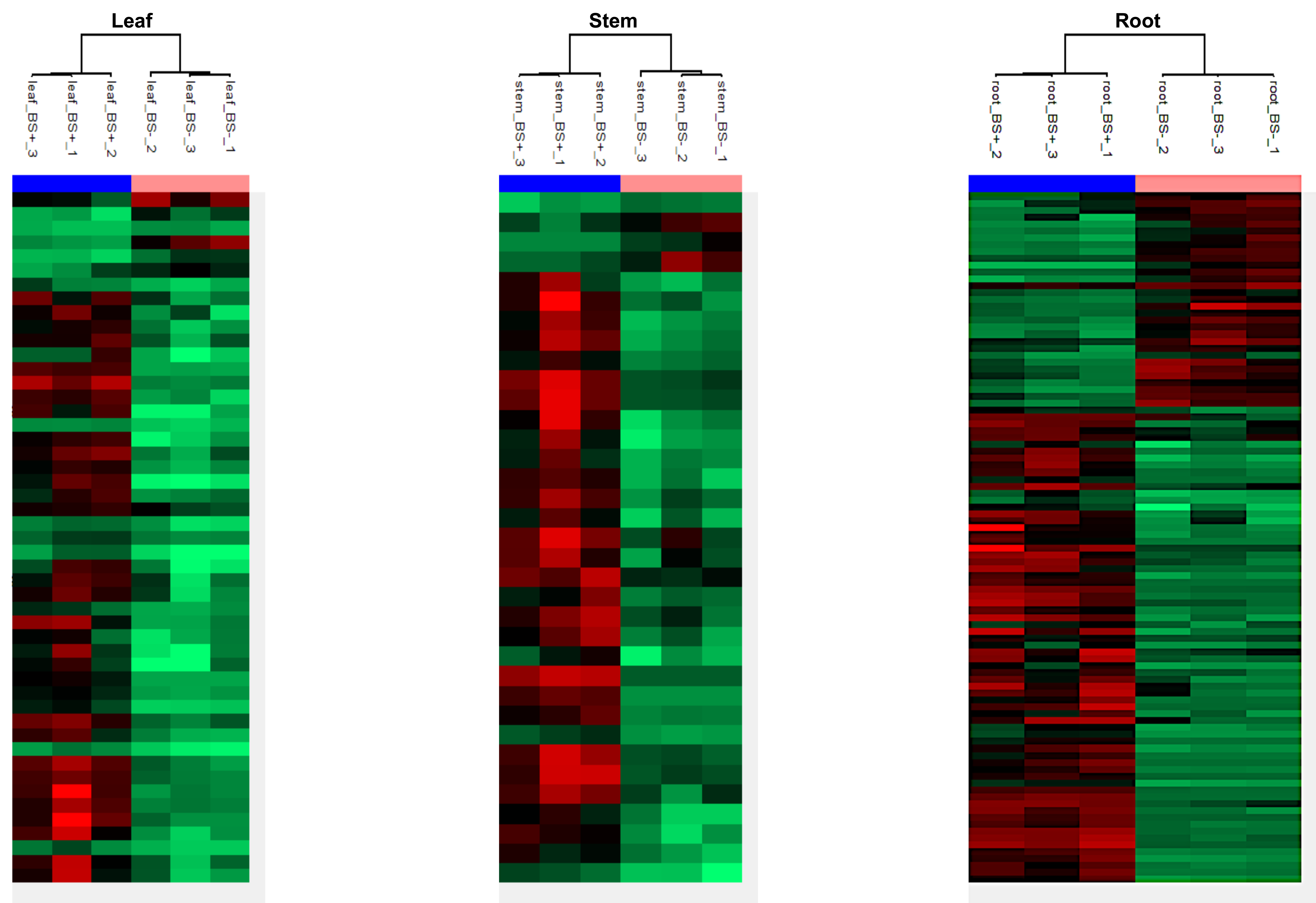

Supplement: Supplementary Figure 3 — Heatmap showing the detected metabolites differently accumulated in leaves, stems and roots of plantlets following treatment by a biostimulant. Grapevine micro-cuttings were transplanted in a solid culture medium supplemented with BS 0.1% (v/v, “BS+”) or water (“BS-” as control). At 4 wpt, plantlets were collected and dissected to fdistinct root, leaf and stem samples. Methanolic extracts were prepared and analyzed by GC-MS. Data were processed using ANOVA (P < 0.05); distance measure used is Pearson’s correlation, and clustering algorithm is average. In red: metabolites more accumulated in BS+ than in BS- organs. [file Image_3.tif]

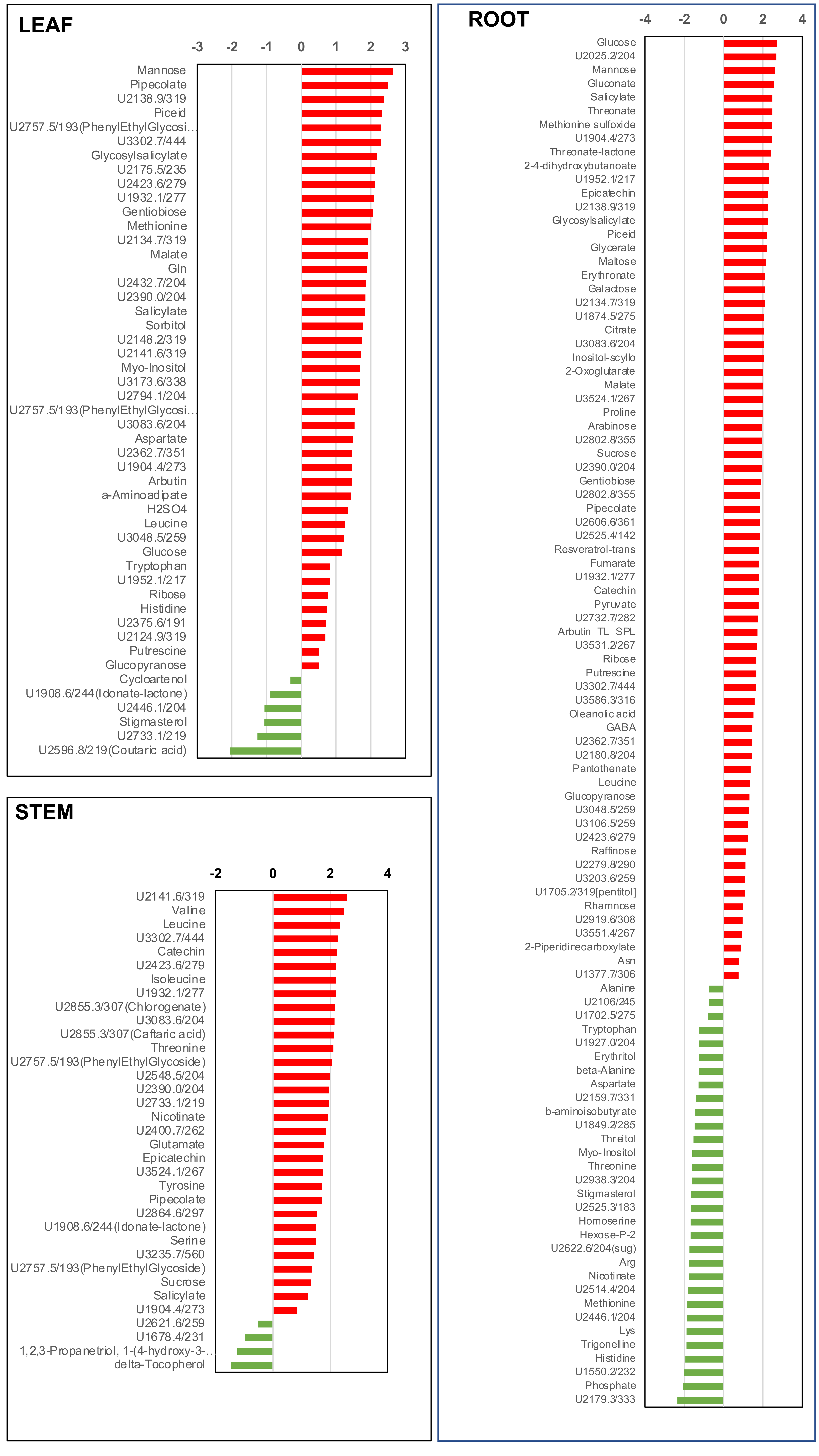

Supplement: Supplementary Figure 4 — List of the metabolites differently accumulated in leaves, stems and roots of BS-treated plantlets, compared to control ones. Grapevine microcuttings were transplanted in a solid culture medium supplemented with BS 0.1% (v/v, “BS+”) or water (“BS-”, as control). At 4 wpt, plantlets were collected and dissected to distinct root, leaf and stem samples. Methanolic extracts were prepared and analyzed by GC-MS. Data were processed using ANOVA (P < 0.05). Red : metabolites more accumulated in BS+ than in BS- organs, In green: metabolites less accumulated in BS+ than in BS- organs. [file Image_4.tif]

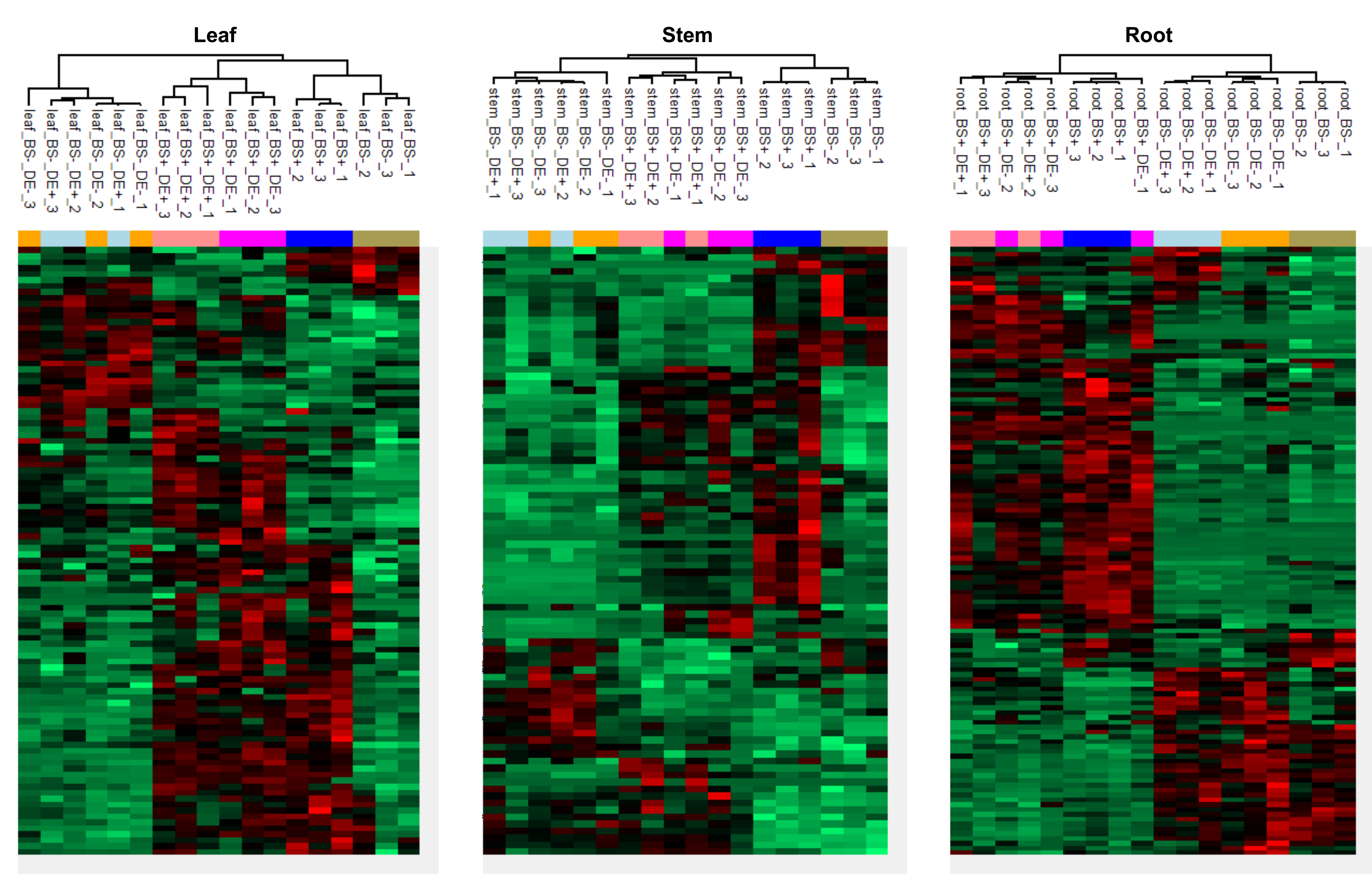

Supplement: Supplementary Figure 5 — Heatmap showing the metabolites differently accumulated in leaves, stems and roots of plantlets following treated by BS or DE. Grapevine micro-cuttings were transplanted in a solid medium supplemented with BS 0.1% (v/v, “BS+”) or water (“BS-”, as control). At 4 wpt, they were divided into two sets: one treated by immersion in a defense elicitor solution (DE+) and the other one in water as control (DE-). Two days later, plantlets were collected and dissected to distinct leaf, stem and root samples. Methanolic extracts were prepared and analyzed by GC-MS. Data were processed using ANOVA (P < 0.05); distance measure used is Pearson’s correlation, and clustering algorithm is average. [file Image_5.tif]
